# Therapeutic targeting of mitochondrial dysfunction in heart failure: a systematic review & meta-analysis of clinical outcomes

**DOI:** 10.3389/fcvm.2026.1824101

**Published:** 2026-06-22

**Authors:** Omer Mohammed, Shabrin Abdul Rasheed, Ananya Arora, Sharon Velamparambil Sunny, Afthab Salam Kanniyan, Shilla Thomas, Basil Wahid Bhat, Venu Pararath Gopalakrishnan, Jeffrey George, Akiva Rosenzveig, Aravinda Nanjundappa, Shelby Kutty

**Affiliations:** 1 Department of General Medicine, Government Medical College, Kozhikode, Kerala, India; 2 Department of Internal Medicine, Basildon University Hospital, Basildon, Essex, United Kingdom; 3 Department of General Medicine, Shri Atal Bihari Vajpayee Medical College & Research Institute, Bangalore, India; 4 International Medical College, Gazipur, Dhaka, Bangladesh; 5 UMass Memorial Health, Worcester, MA, United States; 6 Case Western Reserve University, Cleveland, OH, United States; 7 Cleveland Clinic Foundation, Cleveland Clinic Lerner College of Medicine, Cleveland, OH, United States; 8 Department of Cardiology, BayCare Health System, Clearwater, FL, United States

**Keywords:** coQ10, elamipretide, heart failure, HFpEF, HFrEF, mitochondrial energetics

## Abstract

**Introduction:**

Mitochondrial dysfunction is recognised as a key driver of heart failure (HF) pathophysiology, contributing to oxidative stress, apoptosis, and impaired energy production in cardiomyocytes. Although therapeutic agents aimed at restoring mitochondrial function have demonstrated promise in animal models and early-phase clinical trials, their efficacy in clinical practice remains uncertain. This meta-analysis evaluated the impact of these agents on clinical outcomes in HF patients.

**Methods:**

A systematic literature search was conducted across PubMed, Cochrane Library, ScienceDirect, Google Scholar, and ClinicalTrials.gov for studies published through May 2025. Thirty one studies (24 RCTs and 7 crossover trials) were included in meta-analysis. Standardized mean difference was pooled for changes in left ventricular ejection fraction (LVEF), NYHA class and six-minute walk test (6MWT) distance compared to baseline, and risk ratios (RR) were pooled for heart failure-related hospitalisations, and all-cause mortality.

**Results:**

Interventions significantly improved LVEF compared with baseline (SMD: 0.53; 95% CI: 0.42–0.65; *p* < 0.00001) and control groups (SMD: 0.42; 95% CI: 0.31–0.53). Treatment reduced NYHA functional class (RR: 2.38; 95% CI: 1.48–3.84; *p* = 0.0004), all-cause mortality (RR: 0.62; 95% CI: 0.47–0.82; *p* = 0.0007), and HF-related hospitalizations (RR: 0.60; 95% CI: 0.42–0.85; *p* = 0.004). The certainty of evidence was rated as low across all outcomes owing to substantial heterogeneity and high risk of bias.

**Discussion:**

These findings suggest a potential role for mitochondrial-targeted agents as adjunctive strategies in HF, although the evidence base requires further strengthening through high-quality, adequately powered trials before firm clinical recommendations can be made.

**Systematic Review Registration:**

https://www.crd.york.ac.uk/PROSPERO/view/CRD420251075951, PROSPERO CRD420251075951.

## Introduction

Heart failure represents a critical challenge in contemporary cardiology, with a global prevalence exceeding 56 million cases. This number is expected to rise due to population aging and the increasing burden of cardiovascular risk factors (1). Although current guideline-directed medical therapies effectively modulate neurohormonal activation, they fail to address the underlying metabolic derangements that drive the progression of myocardial dysfunction (2). Persistent high mortality rates exemplify these limitations of existing pharmacologic strategies without significant improvement over the past five years in conjunction with a five-year survival rate for patients with advanced HF remaining below 50% (3).

New therapeutic opportunities have been identified with the discovery of mitochondrial dysfunction as a leading pathophysiological process. Defective oxidative phosphorylation, superfluous production of reactive oxygen species, and injured calcium homeostasis constitute a vicious circle of bioenergetic collapse and cellular apoptosis (4). More recent efforts have rendered it conceptually clear that these mitochondrial aberrations are not incidental findings or side-products, but rather main causes of disease progression, especially in heart failure with reduced EF (HFrEF) (5). This conceptual change has catalysed the generation of precision interventions, which are aimed at salvaging myocardial energetics to the cellular scale.

Several classes of mitochondrial-targeted therapies have emerged as promising approaches. A new tetrapeptide compound, elamipretide, has been identified to have special cardioprotective effects by stabilising cardiolipin, a vital phospholipid required to maintain the integrity of the electron transport chain (6). Clinical trials have further shown that elamipretide may improve left ventricular performance and reduce oxidative harm. Coenzyme Q10 (CoQ10) and its reduced form, ubiquinol, serve as integral components of the electron transport chain and potent antioxidants. The landmark Q-SYMBIO trial additionally demonstrated a significant reduction in mortality with CoQ10 supplementation, supporting its potential for broad clinical applications (7). Metabolic modulators, such as trimetazidine, improve cardiac efficiency during ischemic cardiomyopathy by shifting the utilisation of myocardial substrate away from the less efficient fat oxidation to the more efficient oxygen-utilising glucose metabolism (8). Additionally, polyphenols, such as resveratrol, play a role in the activation of sirtuin pathways, thereby enhancing mitochondrial biogenesis and reducing oxidative damage through multiple molecular mechanisms (9). Preclinical data suggest that these agents may potentially improve diastolic function; however, robust clinical evidence remains limited.

Despite the developments, recent systematic reviews reveal a high degree of heterogeneity in treatment effects across diverse patient subgroups and clinical outcome types (10). While some agents demonstrate marked improvements in ventricular function, others confer only modest clinical benefits. Brown et al. (2022) observed a consistent reduction in hospitalisation rates but no significant effect on mortality (10). These inconsistencies highlight the need to conduct a thorough, high-fidelity assessment of evidence.

This systematic review and associated meta-analysis aim to provide a contemporary comprehensive synthesis of existing literature regarding mitochondrial targeted therapies in HF in improving mechanistic and clinical endpoints in addition to standard care.

## Methods

### Study design

This systematic review and meta-analysis was conducted in accordance with the Cochrane Handbook for Systematic Reviews and reported as per Preferred Reporting Items for Systematic Reviews and Meta-Analyses (PRISMA) statement (Supplementary Table S1) (11). The protocol for this systematic review was registered under PROSPERO (CRD420251075951).

### Selection criteria

We included studies that fulfilled the following criteria: (a) subjects were adults (≥18 years) or children diagnosed with HFrEF or preserved ejection fraction (HFpEF) (b) the use of one or more of the following mitochondria-targeted therapies: elamipretide, coenzyme Q10, trimetazidine, resveratrol, levocarnitine, and cyclosporin (c) RCTs or cross-over trials (d) published in English language (e) had sufficient data on outcomes of interest (Supplementary Table S2). The six agents were selected based on (i) representing mechanistically distinct classes of mitochondrial-targeted intervention (antioxidants, metabolic modulators, mitochondrial peptides, carnitine shuttle facilitators, polyphenolic SIRT1 activators, and MPTP inhibitors), (ii) availability of at least one published clinical trial in a HF population at the time of protocol registration. These outcomes include changes in LVEF, NYHA functional classification, all-cause mortality, HF-related hospitalisation, and six-minute walk test (6-MWT). We excluded prior systematic reviews, meta-analysis, literature reviews, case reports, and editorials on the topic. Studies without sufficient data on outcomes were excluded. We excluded studies that examined the role of these agents in cardiovascular diseases without heart failure (Supplementary Table S2).

### Search strategy and screening for relevant studies

A comprehensive database search was carried out across five major databases: PubMed MEDLINE, Cochrane Library, ScienceDirect, Google Scholar, and ClinicalTrials.gov. using keywords: “heart failure”, “elamipretide”, “resveratrol”, “mitochondria”, “ubiquinol”, “trimetazidine”, “levocarnitine”, “coenzyme Q10” and Boolean operators “AND” or “OR” from inception to May 2025 (Supplementary Table S3). Search results were exported to EndNote Clarivate for reference management. Two independent reviewers (S.A.R and S.T) carried out the primary screening for title and abstract. Full-text articles of potentially relevant studies were retrieved and assessed eligibility by two independent reviewers (S.A.R and S.T). In case of conflict, a third reviewer (O.M) was involved in the resolution, and the final decision of inclusion or exclusion of the text was decided by mutual agreement amongst the reviewers. Additionally, the references of eligible studies were manually searched to identify further relevant articles meeting the inclusion criteria.

### Data extraction

Data extraction was performed independently by two reviewers (A.A and S.V.S), with all entries verified by a third reviewer (O.M) for accuracy. The following study characteristics were extracted: (a) publisher and year of publication (b) number of subjects across study and control group (c) type and dosage of intervention (d) duration of treatment or follow-up (e) type of heart failure (f) geographical location (g) co-existing heart condition. Baseline characteristics of patients extracted from the studies included mean age of patients, number of male and female patients, baseline NYHA Class, baseline LVEF, medication history, including standard of care for heart failure. The following outcome data were extracted: (a) LVEF% compared with controls and compared to pre-existing values as mean ± standard deviation (SD) (b) NYHA class measured as the mean ± SD compared to pre-treatment values, (c) distance walked in meters after 6-MWT compared to controls and pre-treatment levels, (d) improvement in NYHA class score by at least one class following therapy, (e) all-cause mortality, (f) HF-related hospitalisations.

### Risk of bias assessment

An independent reviewer (A.S) carried out the risk of bias assessment for the selected studies using the Cochrane RoB-2 tool for RCTs (12). The risk of bias was classified into three categories: “low risk”, “some concerns”, and “high risk” across five domains: randomization process, deviation from intended intervention, missing outcome data, measurement of outcomes, and selective reporting of outcomes. A sixth domain: bias arising from period and carryover effects, was assessed for crossover trials (13).

### Statistical analysis

The meta-analysis was carried out using RevMan Version 5.4; The Cochrane Collaboration 2020 and Comprehensive Meta-Analysis Software Version 4 (14, 15). We pooled RR for dichotomous variables using using the Mantel-Haenszel statistical method and SMD with the respective 95% confidence interval (CI) for continuous variables using the inverse variance method. Improvement in NYHA class score by at least one class following therapy was pooled as a dichotomous variable. Change in mean NYHA class was pooled as a continuous variable. The heterogeneity of studies was assessed using Chi-square statistics and Higgins I^2^ value (16). Random-effects model was used due to expected clinical heterogeneity (17). We assessed publication bias quantitatively using Egger's linear regression test for outcomes **≥**10 studies and qualitatively using funnel plots for visualisation (18). For outcomes with few studies (<10), interpretation of publication bias is limited. The certainty of evidence and level of recommendation was assessed using the GRADE (Grading of Recommendations Assessment, Development and Evaluation) framework (19). Absolute effects were calculated per 1,000 participants and the number needed to treat (NNT) to compare the effects of these agents across different outcomes. Subgroup analyses were performed according to type of therapeutic agent and HF phenotype. Sensitivity analyses were conducted excluding crossover studies. Multivariate meta-regression models were used to explore potential sources of heterogeneity further. The primary model incorporated study-level covariates such as follow-up duration, mean age, and baseline LVEF. The secondary univariate model categorised each intervention type, using one as a reference in subgroup comparisons. The model's ability to explain between-study variance was assessed via the analog R^2^ statistic. Significance was set at a two-tailed *p* < 0.05.

## Results

**Figure 1:**
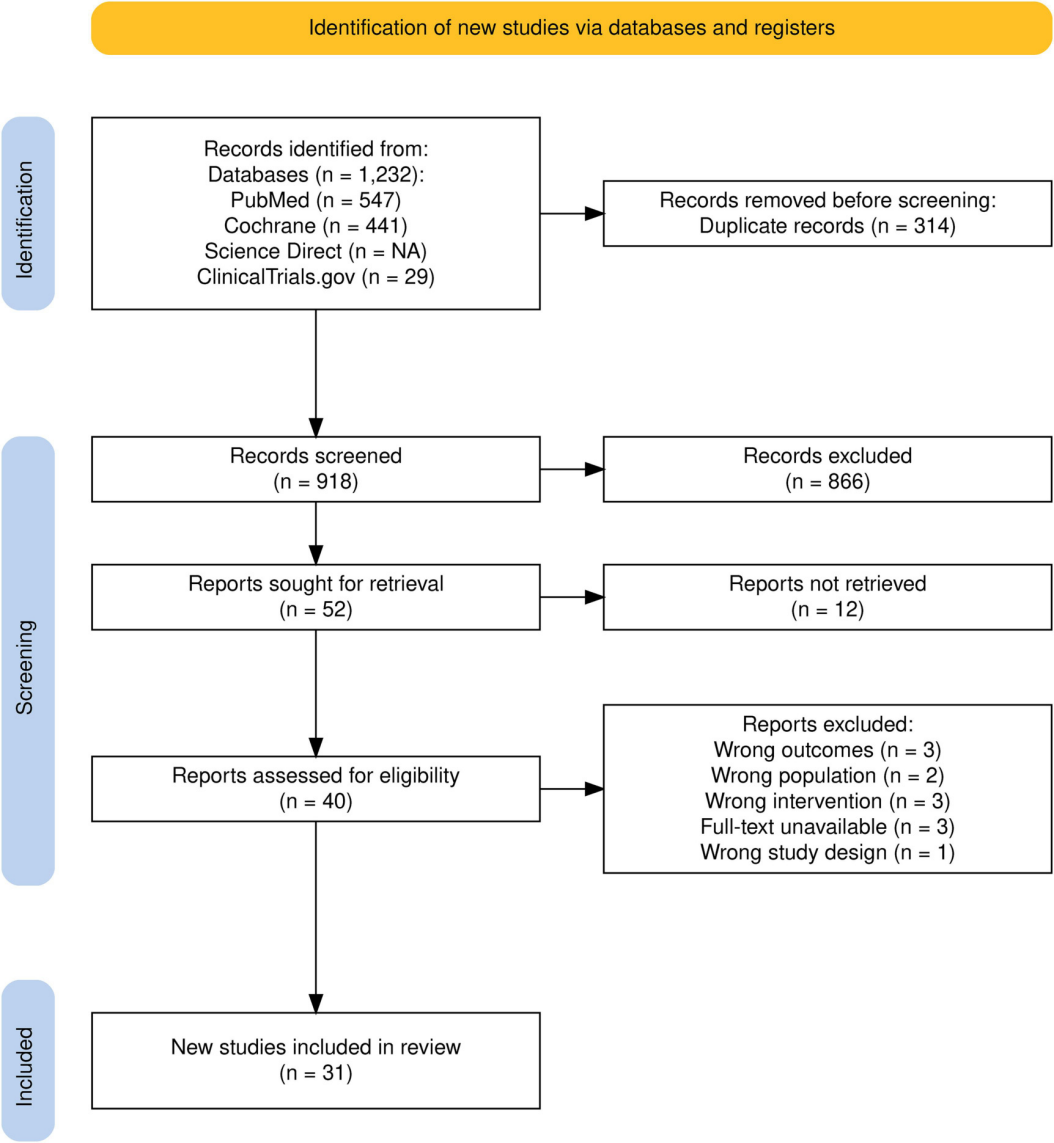
PRISMA study flow diagram.

Our primary search yielded 1232 articles, which were subjected to screening for title and abstract after removal of duplicates. Forty studies underwent full-text screening, out of which we found thirty-one studies with available full text that met our inclusion criteria and was included in the meta-analysis. The screening and selection process is illustrated in Figure 1.

### Quality assessment & risk of bias of included studies

Nine studies were judged to have a low risk of bias, fifteen studies raised some concerns, and seven studies were classified as high risk (Supplementary Figure S1). Overall, most studies demonstrated a low risk of bias in the domains of randomisation and outcome measurement. domain most frequently contributing to a high-risk judgment were deviations from intended interventions, primarily due to the disclosure of intervention to patients and caregivers and the absence of intention-to-treat analyses. The domain of missing outcome data (D3) frequently raised high-risk concerns because patients lost to follow-up or assessors not being blinded to the intervention. Many studies lacked a prior systematic action plan or trial protocol, resulting in “some concern” regarding the risk of selective reporting of results (D5) in most studies. Crossover-specific bias was evaluated for crossover trials and was generally rated as low; however, Langsjoen 2008 and Thrainsdottir 2004 raised some concerns due to insufficient assessment of carryover effects (20, 21).

### Baseline patient characteristics

Twenty-four RCTs and seven crossover trials, published between 1992 and 2023, were included in the meta-analysis, encompassing a total of 2,603 patients. Thirteen studies examined the role of coenzyme Q10 or ubiquinol at doses on heart failure, while nine studies assessed the effect of trimetazidine on subjects with heart failure. L-carnitine was used for intervention in three studies, and the role of elamipretide was assessed in two studies. A study by Daubert 2017 studied varying doses of 4-hour elamipretide infusion on three cohorts (Doses: 0.005 mg/kg/hr, 0.05 mg/kg/hr, 0.25 mg/kg/hr) and designated as Daubert (1), Daubert (2), and Daubert (3), respectively (22). The PROGRESS-HF trial which analysed the impact of elamipretide 4 mg and 40 mg on two cohorts of European HF patients are designated as Butler (1) and Butler (2) respectively (23). Similarly, Thrainsdottir 2004 which evaluated the role of trimezadine in diabetic HF patients, had two treatment periods (21). Their datasets are named as Thrainsdottir (1) and Thrainsdottir (2), respectively, in the analysis. Resveratrol was studied by Magyar 2012 in Caucasian subjects with ischemic heart failure following coronary artery disease (24). Claeys 2019 assessed the efficacy of cyclosporine 2.5 mg/kg in reducing mortality and incidence of hospitalisation in patients with heart failure (25). Kumar 2007 investigated the impact of carniQ-gel (a combination of 2,250 mg/dL-carnitine and 270 mg/d hydrosoluble ubiquinol) in the Indian population (26). Four studies (Samuel 2021, Kinugasa 2020, Bovenkamp 2023, Pierce 2022) involved HFpEF subjects while the remaining twenty-seven studies involved HFrEF subjects (27–30). The range of duration of treatment extended from 4 weeks to 54 weeks. The characteristics of the included studies are given in Table 1.

**Table 1:** Baseline study characteristics of randomised controlled trials evaluating mitochondrial-targeted therapies in patients with heart failure.

Study	Study Design	Location	Mitochondrial agent	Dose (mg)	Follow-up duration	Number MA/C	Heart Failure phenotype	Age MA/C	Male MA/C	Female MA/C	Baseline LVEF	NYHA Class	Other standard medications used
Thrainsdottir 2004 (21)	Double blind, randomised, crossover trials	Sweden	Trimetazidine	20	4 weeks	10/10	HFrEF	67.1 ± 7.2/65.7 ± 9	9/8	1/2	33 ± 10/29 ± 11	II, III	Yes
Kocharian 2009 (49)	Randomised, double-blind, placebo-controlled clinical trial	Iran	Co Q10	2–10	6 months	17/21	HFrEF	6.3 ± 4.5/7.3 ± 5.2	8/11	9/10	29.3 ± 10.2/32.5 ± 10.1	-	Yes
Fragasso 2011 (50)	Single blind randomised study	Italy	Trimetazidine	20	3 months	25/19	HFrEF	70 ± 9/69 ± 12	-	-	35 ± 8/35 ± 7	II, III, IV	Yes
Daubert 2017 (22)	Double-blind, placebo - controlled, ascending-dose trial	Bulgaria	Elamipretide	0.005, 0.05, 0.25^a^	1 week	24/12	HFrEF	61.5/63	18/10	6/2	35.4 ± 8.5, 28.6 ± 12.6, 29.2 ± 8.3^a^	II, III	Yes
Berman 2004 (51)	Randomised, double-blind, controlled trial	Minnesota	Co Q10	60	3 months	32/32	HFrEF		28/28	4/4	-	III, IV	Yes
Morisco 1993 (52)	Randomised, double-blind, placebo controlled parallel group trial	Italy	Co Q10	2	1 year	319/322	HFrEF	67/67	163/157	157/165	-	III, IV	Yes
Khatta 2016 (53)	Randomised, double-blind, controlled trial	USA	Co Q10	200	6 months	23/23	HFrEF	64/64	39/39	7/7	-	III, IV	Yes
Butler 2020 (23)	Randomised, double-blinded, placebo-controlled, multiple-dose study	Europe	Elamipretide	40	28 days	47/24	HFrEF	62 ± 10.8/66.8 ± 9.8	39/15	8/9	31.2 ± 5.7/31.5 ± 7.2	-	Yes
Fragasso 2006 (8)	Randomised open-label study	Italy	Trimetazidine	20	12 months	28/27	HFrEF	64 ± 7/66 ± 7	25/25	3/2	34 ± 7/36 ± 5	II, III, IV	Yes
Mortensen 2014 (54)	Randomised, double-blind, placebo-controlled parallel group trial	Europe, Asia, Australia	Co Q10	100–200	2 years	202/218	HFrEF	62.3 ± 12/62.3 ± 11	154/151	48/67	31 ± 10/31 ± 10	II, III, IV	Yes
Belardinelli 2005 (55)	Double-blind,placebo-controlled cross-over trial	Italy	Co Q10	100	4 weeks	21/21	HFrEF	59 ± 9/59 ± 9	18/18	3/3	37 ± 7/37 ± 7	II, III	Yes
Samuel 2021 (27)	Randomised, double-blind, placebo-controlled trial	Israel	Co Q10	100	4 months	19/20	HFpEF	76 ± 8.97/74.8 ± 9.99	7/11	12/9	59.1 ± 6.1/ 59.3 ± 6.1	II, III, IV	Yes
Hofman-Bang 1995 (56)	Randomised, double-blind, crossover placebo-controlled study	Sweden	Co Q10	100	3 months	69/69	HFrEF	61 ± 10/61 ± 10	-	-	22 ± 10/22 ± 10	II, III, IV	Yes
Mortensen 2019 (7)	Randomised, double-blind, placebo-controlled study	Europe	Co Q10	300	3 months	108/123	HFrEF	65.7 ± 10/64 ± 12	90/87	18/36	33 ± 12/33 ± 12	II, III, IV	Yes
Napoli 2007 (57)	Single-centre, open-label, randomised trial	Italy	Trimetazidine	60	2 years	30/31	HFrEF	67/69	17/18	13/13	30/31	II, III, IV	Yes
Momen 2016 (58)	Randomised, double-blind, placebo-controlled parallel group trial	Bangladesh	Trimetazidine	35	6 months	55/53	HFrEF	58 ± 9.5/59 ± 8.9	45/41	10/12	32.9 ± 6.6/33.1 ± 6.2	II, III, IV	Yes
Pierce 2022 (30)	Randomised, double-blind, placebo-controlled trial	Kansas	Ubiquinol		12 weeks	35/38	HFpEF	-	-	-	51.89/51.04	II, III, IV	Yes
Kumar 2007 (26)	Randomised, double-blind, placebo-controlled trial	India	CarniQ gel	2250/270	12 weeks	29/29	HFrEF	52.9 ± 6.4/50.4 ± 7.7	12/16	17/13	38.8 ± 7.6/39.3 ± 6.7	II, III, IV	Yes
Soongswang 2005 (59)	Randomised, open-label, cross over study	Thailand	Co Q10	3.1 ± 0.6	9 months	15/15	HFrEF	0.6–16.3/0.6–16.3	3/3	12/ 12	30/30	II, III, IV	Yes
Langsjoen 2008 (20)	Randomised, open-label, cross over study	USA	Co Q10	580	20 months	7/7	HFrEF	66/66	2/2	5/5	22/22	IV	Yes
Bovenkamp 2023 (29)	Single-Centre, double-blind, placebo-controlled, randomised, cross-over trial.	Amsterdam	Trimetazidine	20	3 months	8/17	HFpEF	64 ± 10/67 ± 10	3/7	5/10	61/57	II, III	Yes
Belardinelli 2006 (63)	Randomised, double-blind, placebo-controlledcross-over study		Co Q10	100	4 weeks	23/23	HFrEF	59 ± 9/59 ± 9	20/20	3/3	37 ± 7/37 ± 7	II, III, IV	Yes
Wang 2018 (60)	Randomised controlled trial	China	L -carnitine	50- 100	1 year	19/10	HFrEF	-	-	-	-	III, IV	Yes
Claeys 2019 (25)	Multicentre, prospective, double-blinded trial	Belgium	Cyclosporine	2.5	1 year	49/48	HFrEF	63.7 ± 14.4/59.4 ± 12.9	35/5		43 ± 10.3/38.4 ± 13.5	II, III, IV	Yes
Rizos 2000 (31)	Double-blind, placebo-controlled study^b^	Greece	L-carnitine	2000	3 years	37/33	HFrEF	50 ± 14/48 ± 1 2	19/20	23/ 18	27 ± 10/29 ± 11	III, IV	Yes
Winter 2014 (61)	Randomised double-blind study	USA	Trimetazidine	35	6 months	30/30	HFrEF	53 ± 13/57 ± 13	20/21	10/9	30 ± 10/33 ± 10	II, III	Yes
Harjoko 2022 (62)	Double-blind, randomised controlled trial	Indonesia	Trimetazidine	35	12 weeks	12/13	HFrEF	54/60	11/11	1/2	33.3 ± 4.9/35.8 ± 3. 5		Yes
Bohdan 2022 (44)	Randomised, single-centre, open-label, cross-over study	Poland	Trimetazidine	35	6 months	22/23	HFrEF	-	-	-	-	-	Yes
Kinugasa 2022 (28)	Randomised controlled trial		L- carnitine	300 mg	12 months	6/7	HFpEF	-	-	-	58.35 ± 3.71/56.94 ± 6.88	-	Yes
Mancini 1992 (26)	Double-blind, randomised, placebo-controlled trial	Italy	Propionyl L-carnitine	1.5 g	6 months	30/30	HFrEF	59.3 ± 7.3/60.7 ± 6.7 years	23/21	7/9	41.82 + 4.02/41.97 + 4.34%	II–III	Yes
Magyar 2012 (24)	Double-blind, randomised, placebo-controlled trial	Hungary	Resveratrol	10 mg	3 months	20/20	HFrEF	65.3 ± 9.7/67.4 ± 7.7	13/13	7/7	54.8 ± 1.6/52.4 ± 1.6%	-	Yes

This table summarizes the baseline demographic and clinical characteristics (LVEF, NYHA Class, and concomitant standard heart failure medications) of participants enrolled in randomized controlled trials investigating various mitochondrial-targeted agents in heart failure, including coenzyme Q10, trimetazidine, elamipretide, L-carnitine, cyclosporin and ubiquinol.

CoQ10: Coenzyme Q10; LVEF, left ventricular ejection fraction; MA/C, mitochondrial agent/control; mg, milligram; NYHA, New York Heart Association; HFrEF, Heart failure with reserved ejection fraction; HFpEF, Heart failure with preserved ejection fraction.

a Daubert 2017 studied varying doses of 4-hour elamipretide infusion on three cohorts respectively. The rates are in mg·kg^−1.^h^−1.^

b The study was initially blinded for 3 months and then unblinded for the remainder of 3 years.

### Changes in baseline LVEF

**Figure 2:**
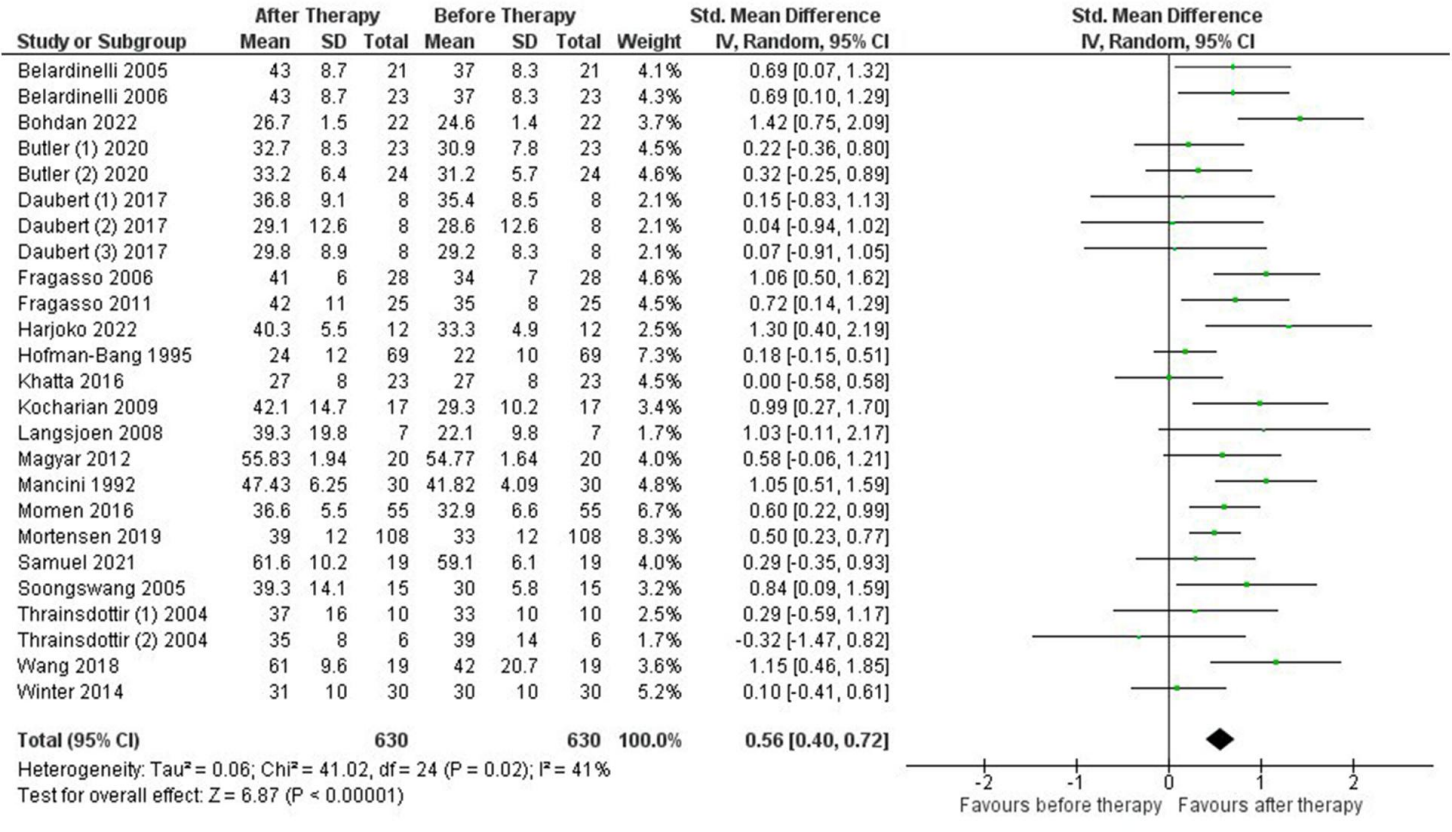
Forest plot comparing mean LVEF after vs. before therapy across included studies using a random-effects model. SD - standard deviation, IV- inverse-variance weighting, CI - confidence intervals.

**Figure 3:**
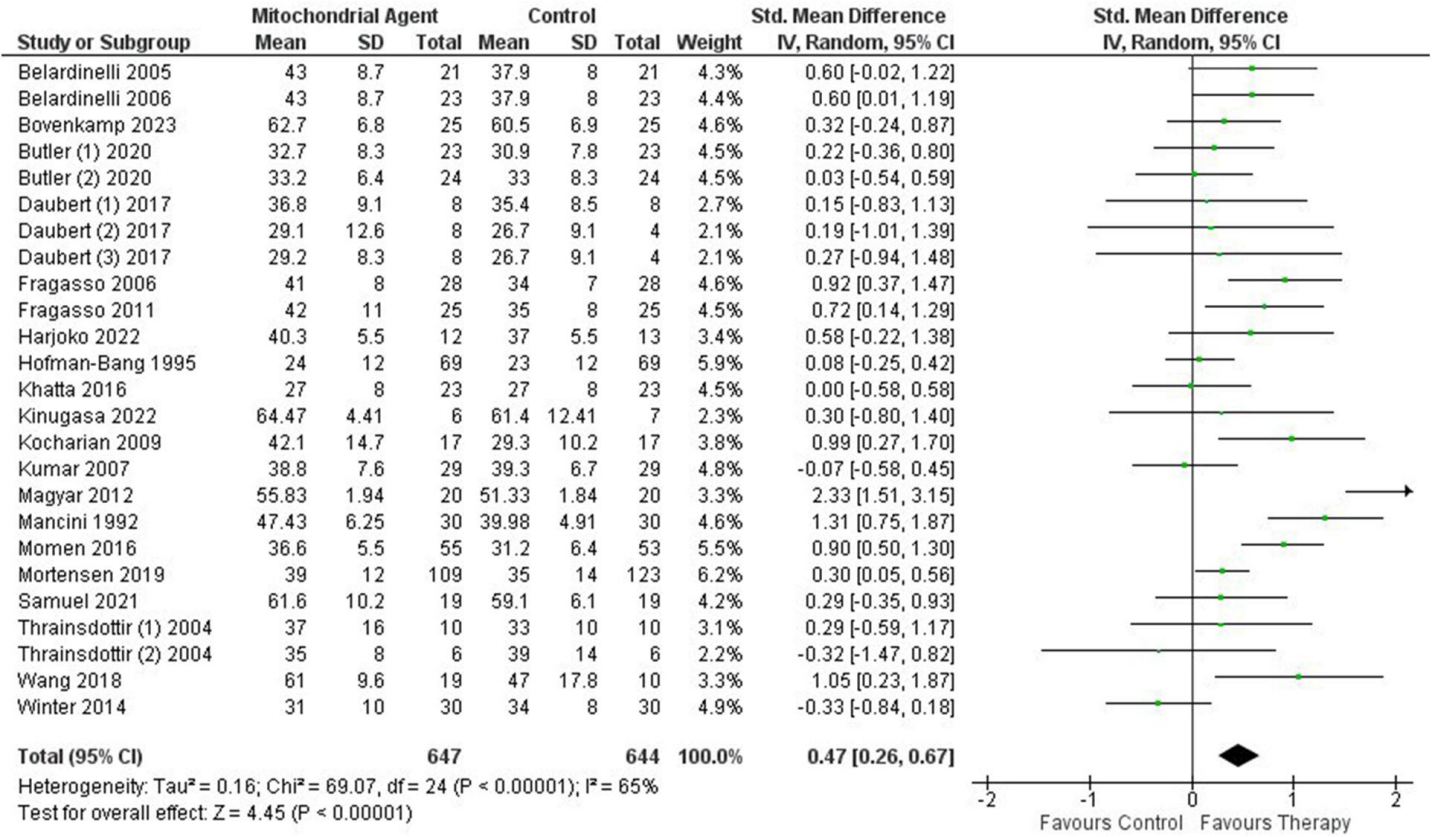
Forest plot comparing mean LVEF after treatment with control group using a random-effects model. SD - standard deviation, IV- inverse-variance weighting, CI - confidence intervals.

Pooled analysis of 25 studies demonstrated a statistically significant improvement in LVEF compared to pre-treatment values (SMD: 0.56; 95% CI: 0.40–0.72; *p* < 0.00001; I^2^ = 41%). and compared to placebo or control (SMD: 0.47; 95% CI: 0.26–0.67; *p* < 0.00001; I^2^ = 65%) Figures 2, 3). Publication bias was not observed for change in LVEF compared to baseline (*p* = 0.45) or to control (*p* = 0.33) (Supplementary Figures S6A, S6B) The GRADE assessment was downgraded due to substantial heterogeneity and high risk of bias of included studies.

### NYHA classification

**Figure 4:**
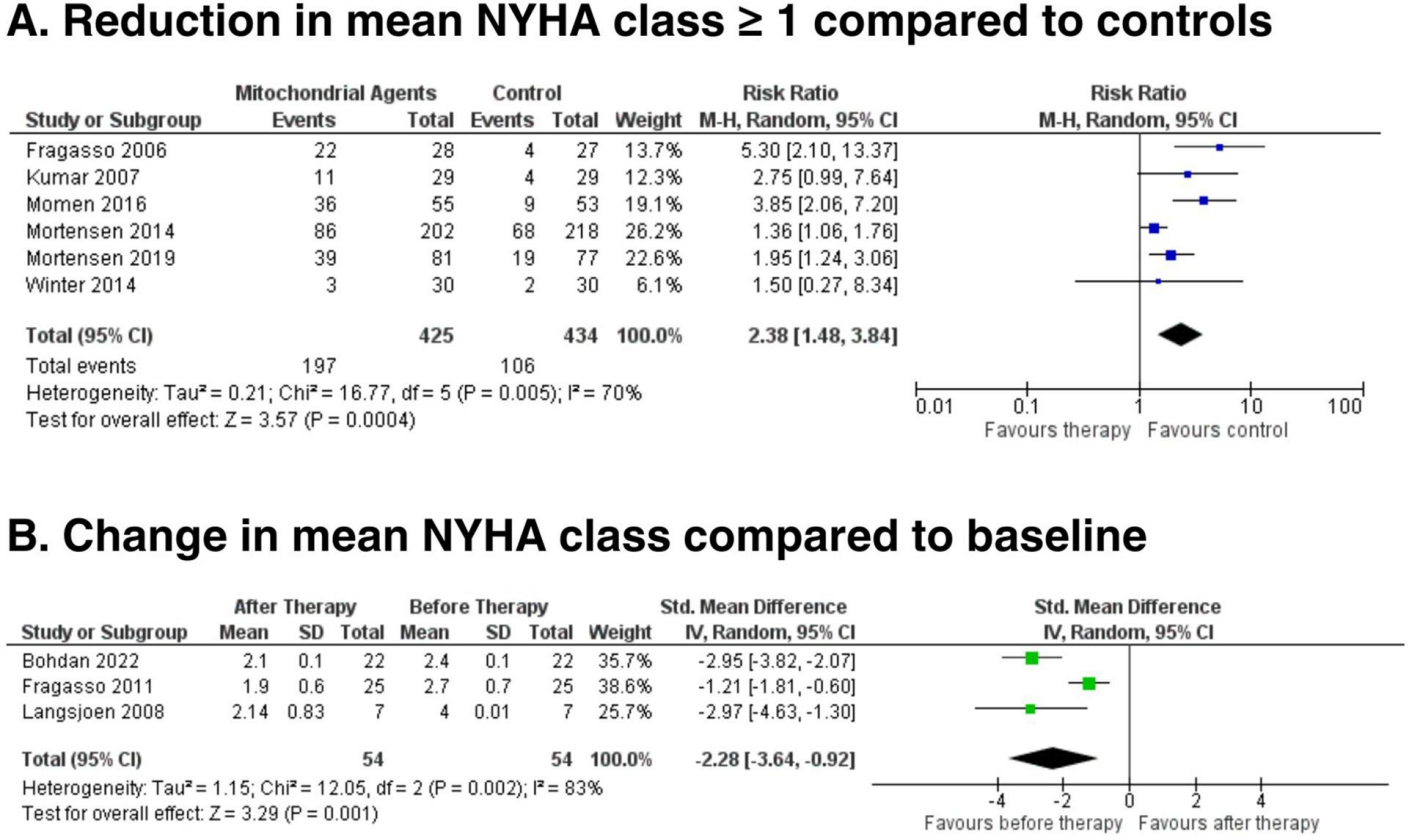
Forest plots evaluating the effect of mitochondrial agents on NYHA class. **(A)** Reduction in NYHA class ≥1 compared with controls, presented as risk ratios with 95% confidence intervals using a random-effects model. **(B)** Change in mean NYHA class from baseline after therapy, presented as standardized mean differences with 95% confidence intervals using a random-effects model. SD - standard deviation, IV- inverse-variance weighting, CI - confidence intervals, M-H: Mantel-Haenszel.

A decrease of at least one NYHA class was considered an indicator of improvement of cardiac function in heart failure subjects. Pooled analysis of six studies showed significant reduction in NYHA class (RR = 2.38; 95% CI: 1.48–3.84; *p* = 0.0004; I^2^ = 70%) compared to controls (Figure 4) with a NNT of 3. Pooled analysis of three studies showed statistically significant reduction in mean NYHA class compared to pre-treatment values (SMD: −2.28; 95% CI: −3.64 to −0.92; *p* = 0.001; I^2^ = 83%) (Figure 4). Funnel plot asymmetry was noted towards negative effect but interpretation is limited by few studies (Supplementary Figures S6C, S6D). The GRADE assessment was downgraded due to substantial heterogeneity and high risk of bias of included studies (Table 2).

**Table 2:** GRADE assessment for the effect of mitochondrial-targeted therapies on clinical outcomes in heart failure patients.

Outcomes	Number of participants (studies)	Certainty of the evidence (GRADE)	Relative effect (95% CI)	Anticipated absolute effects	
				Risk with placebo	Risk difference with mitochondrial agents
Change in baseline LVEF	1,260 (25 RCTs)	⊕⊕⦻⦻ Low	-	-	SMD 0.53 higher (0.42 higher to 0.65 higher)
Change in LVEF compared to controls	1291 (25 RCTs)	⊕⊕⦻⦻ Low	-	-	SMD 0.42 higher (0.31 higher to 0.53 higher)
NYHA Class Improvement	859 (6 RCTs)	⊕⊕⦻⦻ Low	RR 2.38 (1.48 to 3.84)	244 per 1,000	337 more per 1,000 (117 more to 694 more)
Mean NYHA Class	108 (3 RCTs)	⊕⦻⦻⦻ Very low	-	-	SMD 2.28 lower (3.64 lower to 0.92 lower)
Six Minute Walk Test compared to baseline	421 (6 RCTs)	⊕⦻⦻⦻ Very low	-	-	SMD 0.37 SD higher (0.26 lower to 1 higher)
Six Minute Walk Test compared to control	380 (5 RCTs)	⊕⊕⦻⦻ Low	-	-	SMD 0.9 higher (0.06 lower to 1.85 higher)
All-Cause Mortality	1857 (12 RCTs)	⊕⊕⊕⦻ Moderate	RR 0.61 (0.46 to 0.81)	117 per 1,000	45 fewer per 1,000 (63 fewer to 22 fewer)
HF-related Hospitalisations	1567 (8 RCTs)	⊕⊕⦻⦻ Low	RR 0.59 (0.40 to 0.88)	295 per 1,000	121 fewer per 1,000 (177 fewer to 35 fewer)

The table showed low certainty of evidence that mitochondrial-targeted therapies was associated with improvement in clinical and functional outcomes.

CI, confidence interval; RR, risk ratio; SMD, standardised mean difference; HF, heart failure; NYHA, New York Heart Association; RCT, randomised controlled trial; RR, risk ratio; SD, standard deviation.

The risk in the intervention group (and its 95% confidence interval) is based on the assumed risk in the comparison group and the relative effect of the intervention (and its 95% CI).

### Six minute walk test

**Figure 5:**
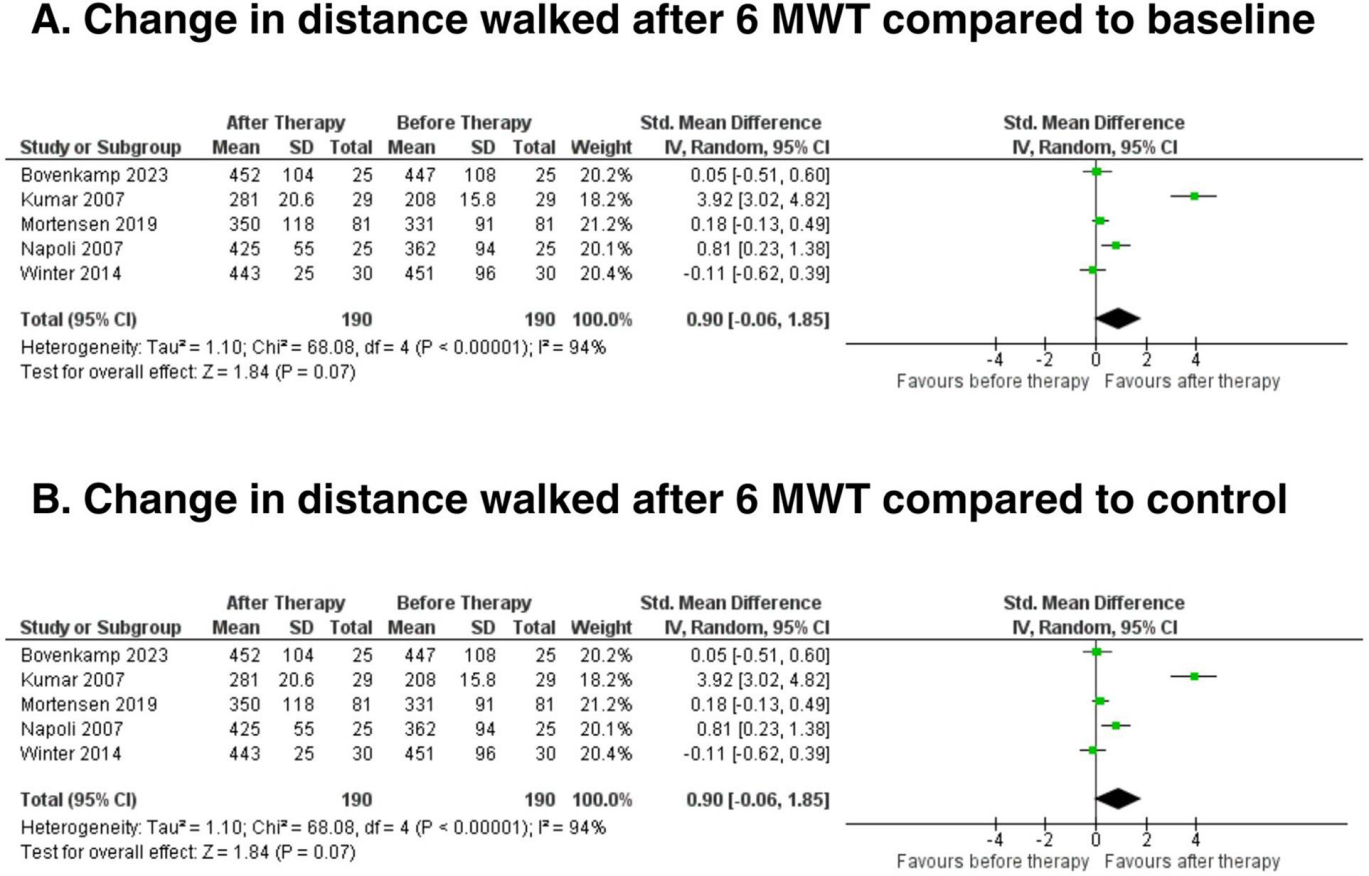
Forest plots evaluating the effect of mitochondrial agents on six-minute walk test **(A)** change in mean distance walked after six-minute walk test compared to baseline **(B)** change in mean distance walked after six-minute walk test following therapy compared to control group. SD - standard deviation, IV- inverse-variance weighting, CI - confidence intervals.

Pooled analysis of six studies showed a comparable mean distance walked after the 6 MWT (SMD: 0.37; 95% CI: −0.26 to 1.00; *p* = 0.25; I^2^ = 89%) compared to controls (Figure 5). These results were consistent when compared to mean distance covered before the start of therapy (SMD: 0.90; 95% CI: −0.06-1.85; *p* = 0.07; I^2^ = 94%) (Figure 5). The GRADE assessment was downgraded due to substantial heterogeneity and high risk of bias of included studies (Table 2). Interpretation of publication bias is limited by few studies (Supplementary Figures S6E, S6F).

### All-cause mortality

**Figure 6:**
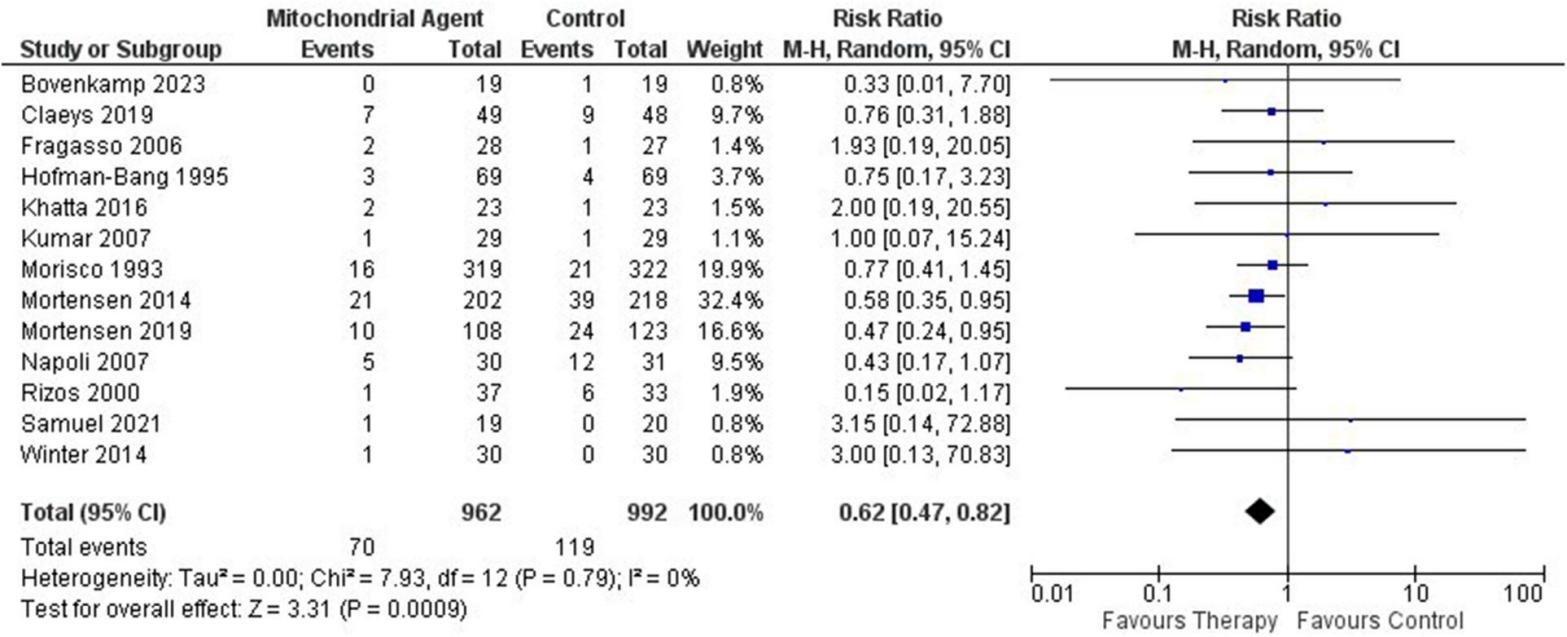
Forest plot comparing risk of all-cause mortality with control group. SD - standard deviation, CI - confidence intervals, M-H: Mantel-Haenszel.

Pooled analysis of 13 studies showed significant reduction in all-cause mortality compared to the control group (RR = 0.62; 95% CI: 0.47–0.82; *p* = 0.0007; I^2^ = 0%) (Figure 6) with NNT of 23. Publication bias was not observed (*p* = 0.28) (Figure S6G). The GRADE assessment was downgraded due to high risk of bias of included studies (Table 2).

### HF-related hospitalisations

**Figure 7:**
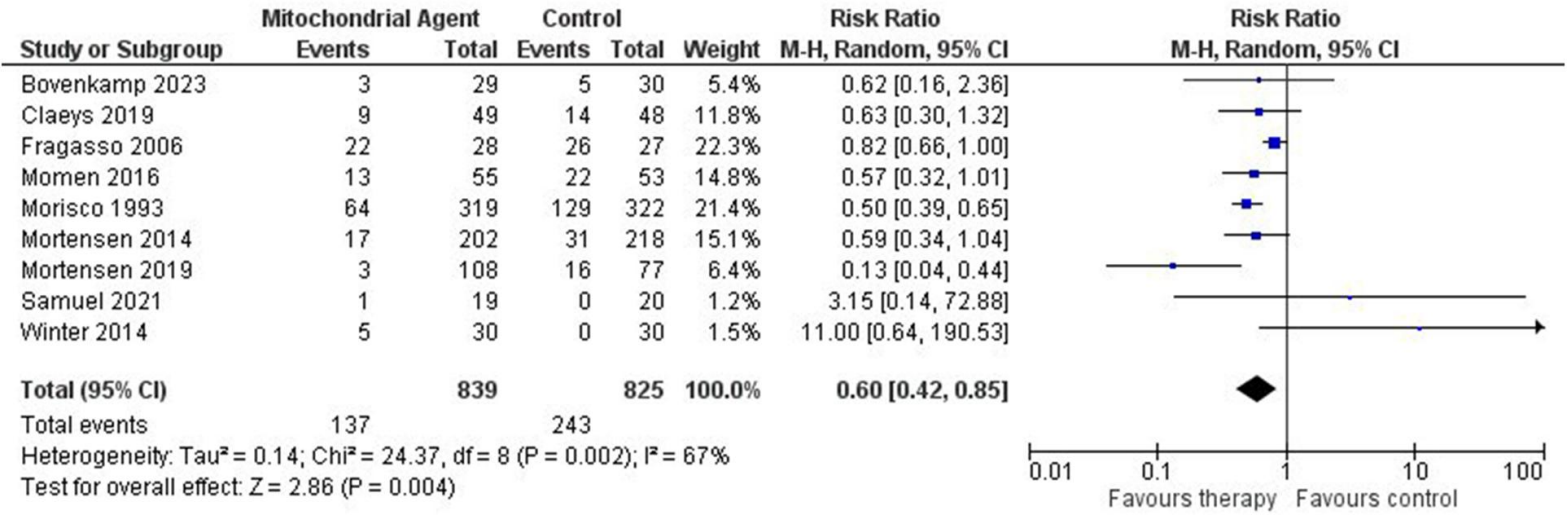
Forest plot comparing risk of heart failure hospitalization with control group. SD - standard deviation, CI - confidence intervals, M-H: Mantel-Haenszel.

Pooled analysis of nine studies demonstrated a statistically significant reduction in HF-related hospitalizations compared to the control group (RR = 0.60; 95% CI: 0.42–0.85; *p* = 0.004; I^2^ = 67%) (Figure 7) with NNT of 9. The GRADE assessment was downgraded due to substantial heterogeneity and high risk of bias of included studies (Table 2). Funnel plot asymmetry was noted towards negative effect but interpretation is limited by few studies (Supplementary Figure S6H).

### Results of subgroup analysis

Elamipretide and resveratrol was not associated with a significant improvement in LVEF compared to baseline (Supplementary Figure S2A). Trimetazidine did not produce a statistically significant reduction in all-cause mortality (RR = 0.57; 95% CI: 0.26–1.26; *p* = 0.17; I^2^ = 0%) (Supplementary Figure S2C) or HF-related hospitalisation (RR = 0.75; 95% CI: 0.49–1.15; *p* = 0.19; I^2^ = 37%) (Supplementary Figure S2D). L-carnitine was not associated with a significant reduction in all-cause mortality (RR = 0.15; 95% CI: 0.02–1.17; *p* = 0.07) (Supplementary Figure S2D). The remaining results were consistent with those of the main analysis (Supplementary Figure S2).

Based on HF phenotype, results of HFrEF subgroup was consistent with main analysis across all outcomes (Supplementary Figure S3). Amongst HFpEF subgroup, there was no statistically significant difference in change in LVEF, six-minute walk test, risk of all-cause mortality or heart failure hospitalizations (Supplementary Figure S3).

### Sensitivity analyses

The results were consistent with the main analysis after exclusion of crossover studies (Supplementary Figure S4).

### Meta-regression

The primary multivariate model including follow-up duration, mean age, and baseline LVEF explained 52% of between-study heterogeneity (Supplementary Table S4, Figure S5). However, older age, longer follow-up duration or higher baseline LVEF was not associated with greater increase in LVEF. Secondary model did not identify any single agent as consistently superior across all models (Supplementary Table S4). However, L-carnitine was associated with a statistically significant improvement in LVEF relative to CoQ10 and elamipretide, while elamipretide was the least effective across models (Supplementary Table S4, Figure S5).

## Discussion

Our meta-analysis demonstrates that mitochondrial-targeted therapy significantly improved LVEF compared to pre-treatment levels and control groups. This improvement is critical, as the degree of LV dysfunction is a strong predictor of mortality (31). There is a clinically meaningful improvement in functional status, as shown by a reduction in NYHA class for every third patient, reflecting fewer functional limitations for patients and enhanced quality of life, both key outcomes in chronic heart failure management. Clinical end-points including all-cause mortality and HF-related hospitalisation were reduced in the therapy group compared to controls with a NNT of 23 and 9 respectively. These findings are similar to the landmark Q-SYMBIO trial, which reported a 42% relative reduction in all-cause mortality with CoQ, and address the ongoing need to reduce rehospitalisation and improve survival in patients receiving guideline-directed medical therapy (7). There was no difference in the distance walked after 6-MWT compared to either their baseline assessment or the control group potentially due to confounding factors like skeletal muscle fraility, comorbid obesity and reduced peak oxygen consumption in HF (27, 30). While results remained consistent on HFrEF subgroup, HFpEF was not associated with clinical or functional improvement. The certainity of evidence is low for seven of the outcomes due to high risk of bias and substantial heterogeneity among the studies.

### Pathophysiological basis for targeting mitochondrial dysfunction in heart failure

Mitochondrial dysfunction in HF contributes to disease pathogenesis through multidimensional roles, including impaired ATP production and increased ROS generation, which lead to oxidative stress and the activation of pro-apoptotic cytochrome c, BAX, and BAK, followed by caspase activation (32–34). These events ultimately result in cardiac myocyte damage and death, thereby promoting disease progression and worsening cardiac function (35).

As adjuncts to conventional heart failure therapy, agents targeting mitochondrial dysfunction address underlying molecular changes within the myocardium and provide a novel and more direct approach to HF treatment (5). Based on their mechanisms of action, these agents can be broadly classified as (a) agents that enhance mitochondrial biogenesis (such as phosphodiesterase type 5 inhibitors, resveratrol, pterostilbene, and other polyphenols), (b) agents that target mitochondrial ROS (including mitoquinone, elamipretide, and coenzyme Q10), (c) agents that modulate specific mitochondrial pathways (such as SIRT1 and SIRT3 activators, including resveratrol), and (d) agents that target the mitochondrial permeability transition pore (MPTP), including cyclosporin A. This meta-analysis evaluated the effects of elamipretide, levocarnitine, coenzyme Q10, resveratrol, cyclosporine, and trimetazidine. Elamipretide acts as a cardiolipin stabilizer, which is an essential component of the inner mitochondrial membrane (7). Coenzyme Q10 serves as a transporter in the electron transport chain and is a potent antioxidant that protects against ROS-induced damage (36). Resveratrol activates sirtuin pathways to enhance mitochondrial biogenesis and modulates multiple mitochondrial signalling mechanisms (37). L-carnitine facilitates the transport of fatty acids into mitochondria, thereby improving energy metabolism in cardiomyocytes and offering potential therapeutic benefits in heart failure (38).

The contrasting therapeutic efficacy of mitochondrial agents between HF phenotypes reflects their distinct pathophysiology. HFrEF represents a primary disease of myocardial energy starvation and cardiomyocyte attrition, whereas HFpEF is predominantly driven by systemic inflammation, extracellular matrix remodeling, and microvascular dysfunction. Because these extra-myocardial structural and inflammatory changes are independent of intrinsic mitochondrial ATP generation capacity, HFpEF heart remains refractory to metabolic resuscitation, limiting efficacy of mitochondrial therapies in this population. Moreover, data on HFpEF is limited (*n* = 4). Hence, cautious interpretation is warranted.

### Comparison with previous meta-analyses

Our findings align with prior meta-analyses, including those by Claxton et al. (2022) and Oleck et al. (2016), which reported that supplementation with Coenzyme Q10 reduced all-cause mortality in HF, and Saadi et al. (2021), Xu et al. (2024), and others that demonstrated improvements in LVEF and functional capacity (36, 39–41). The previous reviews were, however, limited by smaller populations and the exclusion of newer agents like elamipretide or resveratrol, which are included within the scope of this expanded analysis.

In contrast to prior reviews by Zhao et al. (2021) and Nassiri et al. (2024), our meta-analysis did not show mortality or hospitalization benefit with trimetazidine (42, 43). Trimetazidine shifts myocardial energy production from fatty acids to oxygen-efficient glucose oxidation, a mechanism primarily beneficial in ischemic heart disease where oxygen supply is impaired. In contrast, prospective studies on nonischemic heart failure showed no significant improvement in LVEF or quality of life, likely because these patients have preserved oxygen supply, making the metabolic shift less relevant (44). Moreover, beta-blockers already decrease free fatty acid load and inhibit beta-oxidation. Consequently, in trials where nearly all patients (95%) were on beta-blockers, trimetazidine provided diminished returns because the therapeutic window for additional metabolic intervention was already largely addressed by standard therapy (21).

This analysis is the first to include elamipretide that has been recently tested in clinical trials. The PROGRESS-HF trial did not demonstrate significant improvement in LVEF compared to placebo or baseline with subcutaneous elamipretide injections at doses of 4 mg or 40 mg. Similarly, Daubert et.al (2017) examined the role of ascending doses of 4-hour infusion of elamipretide at 0.005 mg/kg/hr, 0.05 mg/kg/hr and 0.25 mg/kg/hr on Bulgarian HF-patients (22) Although the highest infusion dose significantly reduced LV systolic and end-diastolic volumes, there was no significant improvement in LVEF at any dose. Results from our meta-regression indicated that elamipretide was the least effective among the evaluated agents. However, these findings are limited by small sample sizes (71 subjects). Further research is necessary before elamipretide can be recommended as an adjunct to standard therapy in heart failure.

L-carnitine was associated with improved LVEF, consistent with the findings of Zhao et al. (2020), and earlier trials such as Mancini et al. (1992) (45, 46). Rizos et al. (2000), further emphasized the potential benefits of L-carnitine, particularly in patients with dilated cardiomyopathy (31). In our univariate meta-regression model, L-carnitine outperformed coenzyme Q10 and elamipretide for improving LVEF, suggesting its potential value in long-term heart failure management.

The evidence for resveratrol and cyclosporine remains less conclusive. While cyclosporin has shown mechanistic promise in maintaining mitochondrial integrity, its broader immunomodulatory and nephrotoxic effects may limit widespread use in heart failure. Similarly, although resveratrol has proven effective in animal models of heart failure, with some studies suggesting it exerts a protective effect against HFpEF-induced adverse cardiac remodelling, the absence of robust human data precludes definitive conclusions about its clinical utility (47, 48). We included a study by Magyar et.al (2012), that evaluated the role of resveratrol in Hungarian HF-patients with stable coronary artery disease and found non-significant improvement in LVEF and diastolic function (24). Larger clinical trials are needed to clarify the therapeutic role of these agents.

Few prior meta-analyses have evaluated combined formulations, such as Carni Q-gel (L-carnitine plus ubiquinol), which was found to be effective in this study. Such synergy-based strategies may represent a promising direction for future mitochondrial therapies in heart failure.

### Strengths and limitations

This meta-analysis represents one of the most comprehensive assessment to date of mitochondrial-targeted therapies in heart failure, both building upon and extending the scope of earlier analyses. To our knowledge, this is the first meta-analysis to systematically evaluate the impact of newer mitochondrial-based agents, including elamipretide and resveratrol, in the context of heart failure. The inclusion of studies from diverse countries and healthcare settings, including the United States, Europe, South Asia, the Middle East, and Australia, substantially enhances the generalisability of our findings.

Furthermore, subgroup analyses were conducted to evaluate the efficacy of individual agents in improving quality of life and reducing hospitalisations and mortality among patients with heart failure. Multivariate meta-regression models were utilised to identify potential sources of heterogeneity and to evaluate between study variances. The certainty of evidence for each outcome was rigorously assessed using the GRADE framework, thereby improving the interpretability and clinical relevance of the findings. Collectively, these methodological strengths enhance the validity, reproducibility, and applicability of our results.

However, several limitations of our study must be acknowledged. First, most of the crossover trials included in this analysis did not report paired outcomes data. Consequently, these studies were analysed as parallel-group trials, which prevented adjustment for within-subject correlation between intervention periods. This approach may distort estimated variances, and can introduce bias in the precision of effect size estimates. Second, fewer than one-third of the included studies were assessed as having a low risk of bias. As previously described, a substantial proportion of studies lacked a prespecified protocol, involved disclosure of the intervention to patients, or did not perform intention-to-treat analyses, thereby introducing methodological bias. Third, except for all-cause mortality and change in baseline LVEF, most outcomes exhibited moderate-to-severe heterogeneity across studies. Our multivariate meta-regression models, incorporating continuous covariates, explained only 52% of the between-study variance, highlighting the need to explore additional variables, such as agent dosing, heterogeneity in treatment protocols, and the presence of comorbidities or concomitant medications. Data on long-term safety and adverse events were limited and under-reported across studies. Hence, pooling was not possible. Additionally, publication bias may be present due to the exclusion of studies published in languages other than English and the exclusion of unpublished data. Large nutraceutical studies published in other languages were excluded due to lack of translators and language interpreters. Finally, only 13% of studies included HFpEF, thereby limiting the applicability of our findings in this patient population.

Currently, it remains challenging to attribute clinical benefits to any single agent due to heterogeneity in treatment duration, outcome measures, and concurrent therapies. Given that all outcomes were rated as low by the GRADE framework, these findings should be interpreted with appropriate caution. While the overall consistency and direction of results are encouraging and hypothesis-generating, they do not constitute sufficient evidence to support strong clinical recommendations for routine integration of these agents into HF management at this stage. The findings nonetheless support the rationale for further investigation through high-quality, adequately powered RCTs. Notably, a geographic gap exists in the literature: despite the inclusion of studies from diverse regions, none originated from North African or sub-Saharan African populations. This limitation reduces the generalisability of our conclusions. Future research should focus on multicentre, long-term RCT with standardised outcomes particularly in HFpEF and long-term safety, examine dose-response relationships across agents, assess the influence of confounding variables and comorbidities, identify subgroups most likely to benefit from therapy and include participants from vulnerable populations in low and middle-income countries, to guide the future direction of HF clinical practice.

## Conclusion

Therapeutic targeting of mitochondrial dysfunction was associated with improvements in LVEF, NYHA functional class, and reductions in mortality and HF-related hospitalizations in predominantly HFrEF populations, with certainty of evidence across all outcomes. These findings provide preliminary, hypothesis-generating support for further investigation of these agents as adjuncts to guideline-directed medical therapy, particularly for patients with HFrEF who do not achieve optimal outcomes with standard treatment alone. Given the substantial heterogeneity, high risk of bias in included studies, and limited HFpEF representation, high-quality, adequately powered RCTs with standardised outcomes, long-term follow-up, and comprehensive safety reporting are needed to confirm these results and inform future practice.

## Data Availability

The original contributions presented in the study are included in the article/Supplementary Material, further inquiries can be directed to the corresponding author/s.
