# Supplementary material for: Therapeutic targeting of mitochondrial dysfunction in heart failure: a systematic review & meta-analysis of clinical outcomes
Source: Front Cardiovasc Med. 2026 Jun 22;13:1824101. doi: 10.3389/fcvm.2026.1824101 (PMC13333738; doi:10.3389/fcvm.2026.1824101)

**Supplementary Materials**

Supplementary Table S1: PRISMA Checklist

Supplementary Table S2: Inclusion and Exclusion Criteria

Supplementary Table S3: Advanced Search Strategy

Supplementary Table S4: Meta-Regression Results

Supplementary Material: Protocol

Supplementary Figure S1: Risk of Bias Assessment

Supplementary Figure S2: Forest Plots for Subgroup Analyses Based on Type of Therapeutic Agent

Supplementary Figure S3: Forest Plots for Subgroup Analyses Based on Heart Failure Phenotype

Supplementary Figure S4: Forest Plots for Sensitivity Analyses Excluding Crossover Studies

Supplementary Figure S5: Meta-Regression Scatter Plot

Supplementary Figure S6: Funnel Plots for Publication Bias

**Supplementary Table S1: PRISMA Checklist**

| **Section and Topic** | **Item #** | **Checklist item** | **Location where item is reported** |
| --- | --- | --- | --- |
| **TITLE** | | |  |
| Title | 1 | Identify the report as a systematic review. | 1 |
| **ABSTRACT** | | |  |
| Abstract | 2 | Provide an explicit statement of the main objectives, inclusion and exclusion criteria, information sources, risk of bias, methods to synthesise results, participants, limitations of evidence, interpretation of results, primary source of funding, and registration number. | 2 |
| **INTRODUCTION** | | |  |
| Rationale | 3 | Describe the rationale for the review in the context of existing knowledge. | 3 |
| Objectives | 4 | Provide an explicit statement of the objective(s) or question(s) the review addresses. | 4 |
| **METHODS** | | |  |
| Eligibility criteria | 5 | Specify the inclusion and exclusion criteria for the review and how studies were grouped for the syntheses. | 5 |
| Information sources | 6 | Specify all databases, registers, websites, organisations, reference lists and other sources searched or consulted to identify studies. Specify the date when each source was last searched or consulted. | 5 |
| Search strategy | 7 | Present the full search strategies for all databases, registers and websites, including any filters and limits used. | Table S3 |
| Selection process | 8 | Specify the methods used to decide whether a study met the inclusion criteria of the review, including how many reviewers screened each record and each report retrieved, whether they worked independently, and if applicable, details of automation tools used in the process. | 5-6 |
| Data collection process | 9 | Specify the methods used to collect data from reports, including how many reviewers collected data from each report, whether they worked independently, any processes for obtaining or confirming data from study investigators, and if applicable, details of automation tools used in the process. | 6 |
| Data items | 10a | List and define all outcomes for which data were sought. Specify whether all results that were compatible with each outcome domain in each study were sought (e.g. for all measures, time points, analyses), and if not, the methods used to decide which results to collect. | 6 |
|  | 10b | List and define all other variables for which data were sought (e.g. participant and intervention characteristics, funding sources). Describe any assumptions made about any missing or unclear information. | 6 |
| Study risk of bias assessment | 11 | Specify the methods used to assess risk of bias in the included studies, including details of the tool(s) used, how many reviewers assessed each study and whether they worked independently, and if applicable, details of automation tools used in the process. | 6-7 |
| Effect measures | 12 | Specify for each outcome the effect measure(s) (e.g. risk ratio, mean difference) used in the synthesis or presentation of results. | 7 |
| Synthesis methods | 13a | Describe the processes used to decide which studies were eligible for each synthesis (e.g. tabulating the study intervention characteristics and comparing against the planned groups for each synthesis (item #5)). | Table S3 |
|  | 13b | Describe any methods required to prepare the data for presentation or synthesis, such as handling of missing summary statistics, or data conversions. | 7 |
|  | 13c | Describe any methods used to tabulate or visually display results of individual studies and syntheses. | 7 |
|  | 13d | Describe any methods used to synthesize results and provide a rationale for the choice(s). If meta-analysis was performed, describe the model(s), method(s) to identify the presence and extent of statistical heterogeneity, and software package(s) used. | 7 |
|  | 13e | Describe any methods used to explore possible causes of heterogeneity among study results (e.g. subgroup analysis, meta-regression). | 7-8 |
|  | 13f | Describe any sensitivity analyses conducted to assess robustness of the synthesized results. | 7 |
| Reporting bias assessment | 14 | Describe any methods used to assess risk of bias due to missing results in a synthesis (arising from reporting biases). | 7 |
| Certainty assessment | 15 | Describe any methods used to assess certainty (or confidence) in the body of evidence for an outcome. | 7 |
| **RESULTS** | | |  |
| Study selection | 16a | Describe the results of the search and selection process, from the number of records identified in the search to the number of studies included in the review, ideally using a flow diagram. | 8 |
|  | 16b | Cite studies that might appear to meet the inclusion criteria, but which were excluded, and explain why they were excluded. | - |
| Study characteristics | 17 | Cite each included study and present its characteristics. | Table 1 |
| Risk of bias in studies | 18 | Present assessments of risk of bias for each included study. | Figure S1 |
| Results of individual studies | 19 | For all outcomes, present, for each study: (a) summary statistics for each group (where appropriate) and (b) an effect estimate and its precision (e.g. confidence/credible interval), ideally using structured tables or plots. | Table S1 |
| Results of syntheses | 20a | For each synthesis, briefly summarise the characteristics and risk of bias among contributing studies. | 8-9 |
|  | 20b | Present results of all statistical syntheses conducted. If meta-analysis was done, present for each the summary estimate and its precision (e.g. confidence/credible interval) and measures of statistical heterogeneity. If comparing groups, describe the direction of the effect. | 9-12 |
|  | 20c | Present results of all investigations of possible causes of heterogeneity among study results. | 11-12 |
|  | 20d | Present results of all sensitivity analyses conducted to assess the robustness of the synthesized results. | - |
| Reporting biases | 21 | Present assessments of risk of bias due to missing results (arising from reporting biases) for each synthesis assessed. | 9-11 |
| Certainty of evidence | 22 | Present assessments of certainty (or confidence) in the body of evidence for each outcome assessed. | Table 2 |
| **DISCUSSION** | | |  |
| Discussion | 23a | Provide a general interpretation of the results in the context of other evidence. | 12-13 |
|  | 23b | Discuss any limitations of the evidence included in the review. | 16-17 |
|  | 23c | Discuss any limitations of the review processes used. | 16-17 |
|  | 23d | Discuss implications of the results for practice, policy, and future research. | 12-18 |
| **OTHER INFORMATION** | | |  |
| Registration and protocol | 24a | Provide registration information for the review, including register name and registration number, or state that the review was not registered. | 5 |
|  | 24b | Indicate where the review protocol can be accessed, or state that a protocol was not prepared. | S12-15 |
|  | 24c | Describe and explain any amendments to information provided at registration or in the protocol. | S12-15 |
| Support | 25 | Describe sources of financial or non-financial support for the review, and the role of the funders or sponsors in the review. | 18 |
| Competing interests | 26 | Declare any competing interests of review authors. | 18-19 |
| Availability of data, code, and other materials | 27 | Report which of the following are publicly available and where they can be found: template data collection forms; data extracted from included studies; data used for all analyses; analytic code; any other materials used in the review. | - |

**Supplementary Table S2: Inclusion and Exclusion Criteria**

| **INCLUSION CRITERIA** | **EXCLUSION CRITERIA** |
| --- | --- |
| **Population:** Adults (≥18 years) or children diagnosed with heart failure with reduced ejection fraction (HFrEF) or preserved ejection fraction (HFpEF) | Patients with cardiovascular diseases other than heart failure. |
| **Study Design:** Randomised Controlled Trials, Crossover Trials | Systematic Reviews, Meta-Analysis, Literature Review, Editorials, Case Reports |
| **Language:** English | Published in languages other than English |
| **Intervention:** Mitochondrial-targeted agents (elamipretide, coenzyme Q10, trimetazidine, resveratrol, levocarnitine, cyclosporin) |  |
| **Comparator:** Placebo or standard of care in HF |  |
| **Outcomes:** Left Ventricular Ejection Fraction (LVEF), NYHA functional classification, six-minute walk test (6-MWT), HF-related hospitalizations, all-cause mortality | Studies without sufficient data on outcomes of interest. |
| **Period:** Studies published from inception |  |

**Supplementary Table S3: Advanced Search Strategy**

Databases included:

 1. PubMed MEDLINE

 2. Cochrane Library

 3. Science Direct

 4. Google Scholar

 5. ClinicalTrials.gov.

| Database | Search Strategy | Results |
| --- | --- | --- |
| PubMed MEDLINE | (("Heart Failure"[Mesh] OR "heart failure"[tiab] OR "cardiac failure"[tiab] OR "congestive heart failure"[tiab]) AND ("Mitochondria"[Mesh] OR "mitochondrial"[tiab] OR "mitochondria-targeted"[tiab] OR "mitochondria-directed"[tiab]) AND ("Therapeutics"[Mesh] OR "therapy"[Subheading] OR "treatment"[tiab] OR "drug therapy"[tiab] OR "intervention"[tiab]) AND ("Elamipretide"[tiab] OR "SS-31"[tiab] OR "Bendavia"[tiab] OR "MitoQ"[tiab] OR "Mito-TEMPO"[tiab] OR "Coenzyme Q10"[tiab] OR "Ubiquinone"[tiab] OR "Idebenone"[tiab] OR "Szeto-Schiller peptide"[tiab] OR "EPI-743"[tiab] OR "Nicotinamide Riboside"[tiab] OR "NR"[tiab] OR "Nicotinamide Mononucleotide"[tiab] OR "NMN"[tiab])),(Elamipretide) AND (Heart Failure) | 133 |
|  | (Elamipretide) AND (Heart Failure) | 43 |
|  | (CoenzymeQ10) AND (Heart Failure) | 3 |
|  | (Resveratrol) AND (Heart Failure) | 180 |
|  | (Trimetazidine) AND (Heart Failure) | 188 |
| Cochrane Library | (Elamipretide OR cyclosporin OR resveratrol OR levocarnitine OR Coenzyme Q10 OR trimetazidine) AND (heart Failure OR cardiac Failure OR congestive cardiac failure) | 441 |
| Science Direct | (Elamipretide OR cyclosporin OR resveratrol OR levocarnitine OR Coenzyme Q10) AND (heart Failure OR cardiac Failure OR congestive cardiac failure) | 115 |
| Google Scholar* | (Elamipretide OR cyclosporin OR resveratrol OR levocarnitine OR Coenzyme Q10) AND (heart Failure OR cardiac Failure OR congestive cardiac failure) | 100 |
| ClinicalTrials.gov. | (Elamipretide OR cyclosporin OR resveratrol OR levocarnitine OR Coenzyme Q10) AND heart failure | 29 |

* Only the first 100 articles of Google Scholar in order of relevance were screened to avoid redundancy

**Supplementary Table S4: Meta-Regression Results**

**Multivariate Regression Model for LVEF using Type of Agent as Covariates**

| Reference Agent | Intercept | Agent Compared | Coefficient (vs Ref) | 95% CI | P-value | Direction | R² analog | Model Q (p) |
| --- | --- | --- | --- | --- | --- | --- | --- | --- |
| **Coenzyme Q10** | 0.4981 | Elamipretide | -0.2958 | -0.7489 to 0.1573 | 0.2007 | No significant difference | 0.35 | 8.75 (0.0675) |
|  | 0.4981 | L-Carnitine | 0.6124 | 0.0424 to 1.1823 | 0.0352 | Higher LVEF | 0.35 | 8.75 (0.0675) |
|  | 0.4981 | Resveratrol | 0.0920 | -0.7005 to 0.8845 | 0.8200 | No significant difference | 0.35 | 8.75 (0.0675) |
|  | 0.4981 | Trimetazidine | 0.1945 | -0.1605 to 0.5495 | 0.2829 | No significant difference | 0.35 | 8.75 (0.0675) |
| **Elamipretide** | 0.2023 | Coenzyme Q10 | 0.2958 | -0.1573 to 0.7489 | 0.2007 | No significant difference | 0.35 | 8.75 (0.0675) |
|  | 0.2023 | L-Carnitine | 0.9081 | 0.2587 to 1.5576 | 0.0061 | Higher LVEF | 0.35 | 8.75 (0.0675) |
|  | 0.2023 | Resveratrol | 0.3878 | -0.4636 to 1.2393 | 0.3720 | No significant difference | 0.35 | 8.75 (0.0675) |
|  | 0.2023 | Trimetazidine | 0.4903 | 0.0181 to 0.9625 | 0.0418 | Higher LVEF | 0.35 | 8.75 (0.0675) |
| **L-Carnitine** | 1.1104 | Coenzyme Q10 | -0.6124 | -1.1823 to -0.0424 | 0.0352 | Lower LVEF | 0.35 | 8.75 (0.0675) |
|  | 1.1104 | Elamipretide | -0.9081 | -1.5576 to -0.2587 | 0.0061 | Lower LVEF | 0.35 | 8.75 (0.0675) |
|  | 1.1104 | Resveratrol | -0.5203 | -1.4393 to 0.3987 | 0.2671 | No significant difference | 0.35 | 8.75 (0.0675) |
|  | 1.1104 | Trimetazidine | -0.4179 | -1.0032 to 0.1674 | 0.1617 | No significant difference | 0.35 | 8.75 (0.0675) |
| **Resveratrol** | 0.5901 | Coenzyme Q10 | -0.0920 | -0.8845 to 0.7005 | 0.8200 | No significant difference | 0.35 | 8.75 (0.0675) |
|  | 0.5901 | Elamipretide | -0.3878 | -1.2393 to 0.4636 | 0.3720 | No significant difference | 0.35 | 8.75 (0.0675) |
|  | 0.5901 | L-Carnitine | 0.5203 | -0.3987 to 1.4393 | 0.2671 | No significant difference | 0.35 | 8.75 (0.0675) |
|  | 0.5901 | Trimetazidine | 0.1025 | -0.7011 to 0.9060 | 0.8027 | No significant difference | 0.35 | 8.75 (0.0675) |
| **Trimetazidine** | 0.6926 | Coenzyme Q10 | -0.1945 | -0.5495 to 0.1605 | 0.2829 | No significant difference | 0.35 | 8.75 (0.0675) |
|  | 0.6926 | Elamipretide | -0.4903 | -0.9625 to -0.0181 | 0.0418 | Lower LVEF | 0.35 | 8.75 (0.0675) |
|  | 0.6926 | L-Carnitine | 0.4179 | -0.1674 to 1.0032 | 0.1617 | No significant difference | 0.35 | 8.75 (0.0675) |
|  | 0.6926 | Resveratrol | -0.1025 | -0.9060 to 0.7011 | 0.8027 | No significant difference | 0.35 | 8.75 (0.0675) |

**Meta-Regression for Continuous Covariates and LVEF**

| Covariate | **Coefficient** | **95% CI** | **P-value** | **Direction** | **R² analog** | **Model Q (p)** |
| --- | --- | --- | --- | --- | --- | --- |
| Follow-up Duration | 0.0103 | -0.0013 to 0.0219 | 0.0826 | Non-significant increase | 0.52 | Q = 7.45, p = 0.0588 |
| Mean Age | -0.0064 | -0.0198 to 0.0070 | 0.3487 | Non-significant increase |  |  |
| Baseline LVEF | 0.0124 | -0.0070 to 0.0317 | 0.2102 | Non-significant increase |  |  |

**Supplementary Material: Protocol**

1. **Title:**

   Therapeutic targeting of mitochondrial dysfunction in heart failure: A systematic review and meta-analysis of clinical outcomes
2. **Review objectives:**

   Do mitochondrial targeted therapies improve outcomes such as LVEF, NYHA classification, hospitalization and mortality in patients with heart failure?
3. **Background and Rationale:**

   Mitochondrial dysfunction is increasingly being recognized as a central contributor to heart failure pathophysiology. While therapeutic agents targeting to restore mitochondrial function like elamipretide, resveratrol, ubiquinol, and trimetazidine have shown promise in animal models and early-phase human trials, their clinical efficacy remains uncertain. The objective of this meta-analysis is to assess the efficacy of these therapeutic agents in improving the outcomes of people with heart failure.
4. **Inclusion Criteria:**

**Population**: Studies involving adults or children with HFrEF or HFpEF

**Study Design:** Randomised Controlled trials, Cross-over trials

**Intervention:** Mitochondrial-targeted agents (elamipretide, coenzyme Q10, trimetazidine, resveratrol, levocarnitine)

**Comparator:** Placebo or standard of care in Heart Failure

**Outcomes:** changes in LVEF, improvement in NYHA class, six-minute walk test, death, risk of hospitalization, cardiac output

**Period:** Studies published from inception

# **Exclusion Criteria:**

- Systematic reviews, meta-analysis, literature reviews, case reports, and editorials
- Studies without sufficient data on outcomes
- Studies published in languages other than English
- Studies not involving heart failure

# **Information Sources:**

- PubMed
- Cochrane Library
- ScienceDirect
- Google Scholar
- ClinicalTrials. Gov

1. **Search Strategy:**

   7.1. PubMed**(Including MEDLINE) :**

(("Heart Failure"[Mesh] OR "heart failure"[tiab] OR "cardiac failure"[tiab] OR "congestive heart failure"[tiab]) AND ("Mitochondria"[Mesh] OR "mitochondrial"[tiab] OR "mitochondria-targeted"[tiab] OR "mitochondria-directed"[tiab]) AND ("Therapeutics"[Mesh] OR "therapy"[Subheading] OR "treatment"[tiab] OR "drug therapy"[tiab] OR "intervention"[tiab]) AND ("Elamipretide"[tiab] OR "SS-31"[tiab] OR "Bendavia"[tiab] OR "MitoQ"[tiab] OR "Mito-TEMPO"[tiab] OR "Coenzyme Q10"[tiab] OR "Ubiquinone"[tiab] OR "Idebenone"[tiab] OR "Szeto-Schiller peptide"[tiab] OR "EPI-743"[tiab] OR "Nicotinamide Riboside"[tiab] OR "NR"[tiab] OR "Nicotinamide Mononucleotide"[tiab] OR "NMN"[tiab])),(Elamipretide) AND (Heart Failure),(CoenzymeQ10) AND (Heart Failure) OR (Levocarnitine) AND (Heart Failure) OR(Resveratrol) AND (Heart Failure) OR(Trimetazidine) AND (Heart Failure) OR (Cyclosporin) AND (Heart Failure)

7.2 **Cochrane Library:**

(Elamipretide OR cyclosporin OR resveratrol OR levocarnitine OR Coenzyme Q10 OR trimetazidine) AND (heart Failure OR cardiac Failure OR congestive cardiac failure)

7.3 **Google Scholar:**

(Elamipretide OR cyclosporin OR resveratrol OR levocarnitine OR Coenzyme Q10) AND (heart Failure OR cardiac Failure OR congestive cardiac failure)

7.4 **ScienceDirect:**

(Elamipretide OR cyclosporin OR resveratrol OR levocarnitine OR Coenzyme Q10) AND (heart Failure OR cardiac Failure OR congestive cardiac failure)

7.5 **ClinicalTrials.gov:**

(Elamipretide OR cyclosporin OR resveratrol OR levocarnitine OR Coenzyme Q10) AND heart failure

1. **Data Extraction:**

Two reviewers independently extracted data on:

- **Study characteristics:** Publication Year, Journal, Geographic Location, Type of Mitochondrial-Targeted Therapeutic Agent and Dose, Duration of Follow-up, Type of Heart Failure, Co-existing heart diseases
- **Patient Characteristics**: Number, Mean Age, Sex, Baseline NYHA Class, Baseline LVEF, Medication history
- **Outcomes:** changes in LV Ejection Fraction (mean+-SD), Six-Minute Walk Test, NYHA Classification (mean+-SD), improvement in NYHA Class, number of HF-related hospitalizations, all-cause mortality.

1. **Risk of Bias Assessment:**

- RCTs: Cochrane Risk of Bias 2.0.

The risk of bias was assessed across five domains: randomization process, deviation from intended intervention, missing outcome data, measurement of outcomes, and selective reporting of outcomes.

# **Data Synthesis:**

- **Software:** Review Manager 5.4
- **Continuous data:** standardized mean difference (SMD) with the respective 95% confidence interval (CI)
- **Dichotomous data:**  pooled risk ratio (RR) with 95% confidence intervals
- **Heterogeneity Assessment**: Chi-square statistics, Higgins I² value
- **Publication bias:** Quantitatively using Egger’s linear regression test and qualitatively using funnel plots for visualization.

1. **Subgroup Analysis :**

- Based on different types of therapeutic agents.

1. **Multivariate meta-regression models:**

- Multivariate meta-regression models will be employed to explore potential sources of heterogeneity.
- Multivariate covariates: Mean Age, Duration of Follow-up, Baseline LVEF, Type of Agent

1. **PROSPERO Registration Date:** 19 June 2025
2. **Conflicts of Interest:** None declared
3. **Ethics and Dissemination:**

As this study is a secondary analysis of published de-identified data, IEC/IRB approval is not required.

Findings will be published in a peer-reviewed journal. The review will be reported in accordance with the PRISMA 2020 guidelines

1. **Source of Funding:**

None declared.

1. **Review Team members:**

Omer Mohammed
Shabrin Abdul Rasheed
Sharon V.S
Shilla Thomas
Ananya Arora
Afthab Salam

Basil Whad Bhat

Venu Pararath Gopalakrishnan

Jeffrey George

Akiva Rosenzveig

Aravinda Nanjundappa

Shelby Kutty

Note: The initial protocol did not include assessment of certainty of evidence using GRADE framework. However, the team members mutually decided to evaluate certainty of evidence for each outcome using the GRADE framework once the meta-analysis was complete, to enhance the interpretability and clinical relevance of the conclusions.

**Supplementary Figure S1: Risk of Bias Assessment**

Figure S1A: Risk of Bias Assessment of crossover trials using Cochrane RoB 2.0 tool


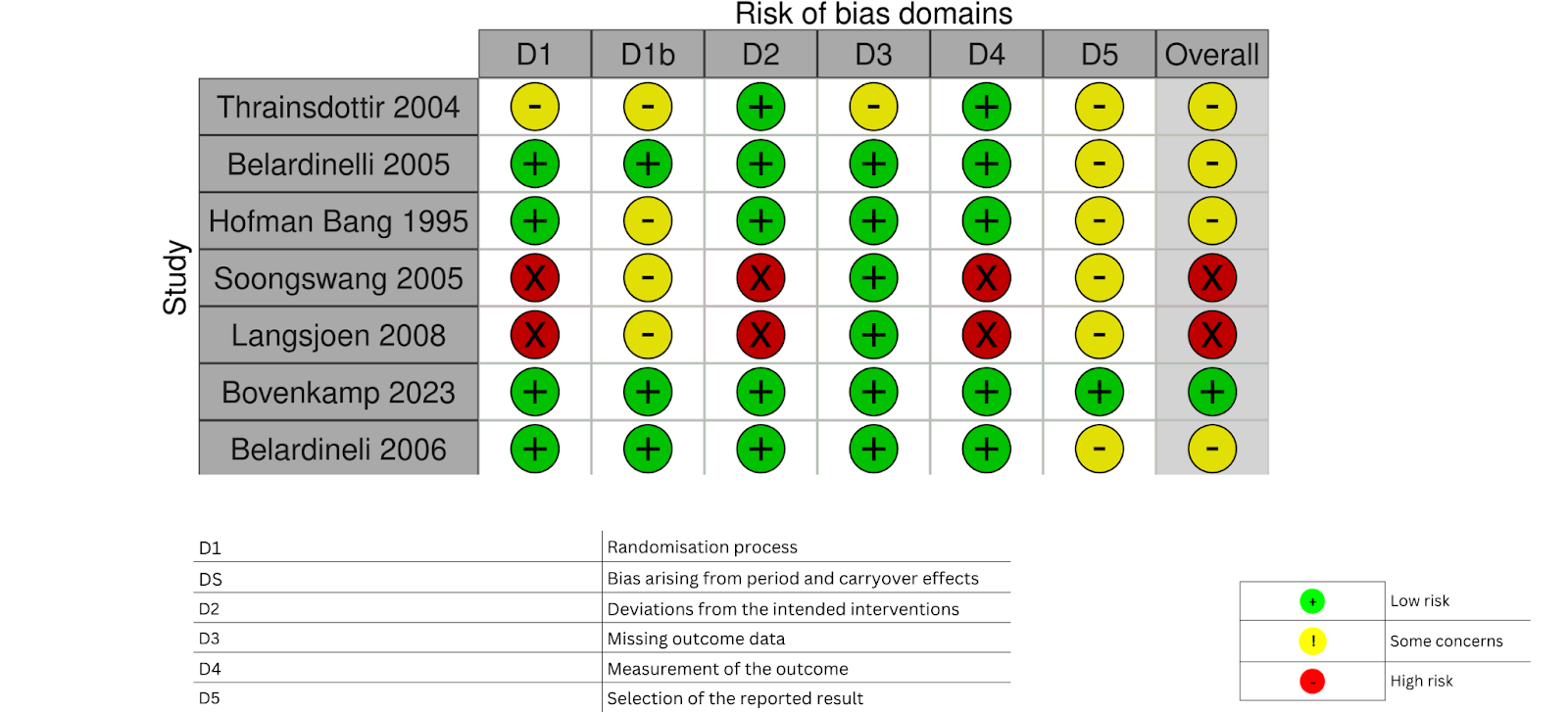


Figure S1B: Risk of Bias Assessment of non-crossover RCTs using Cochrane RoB 2.0 tool


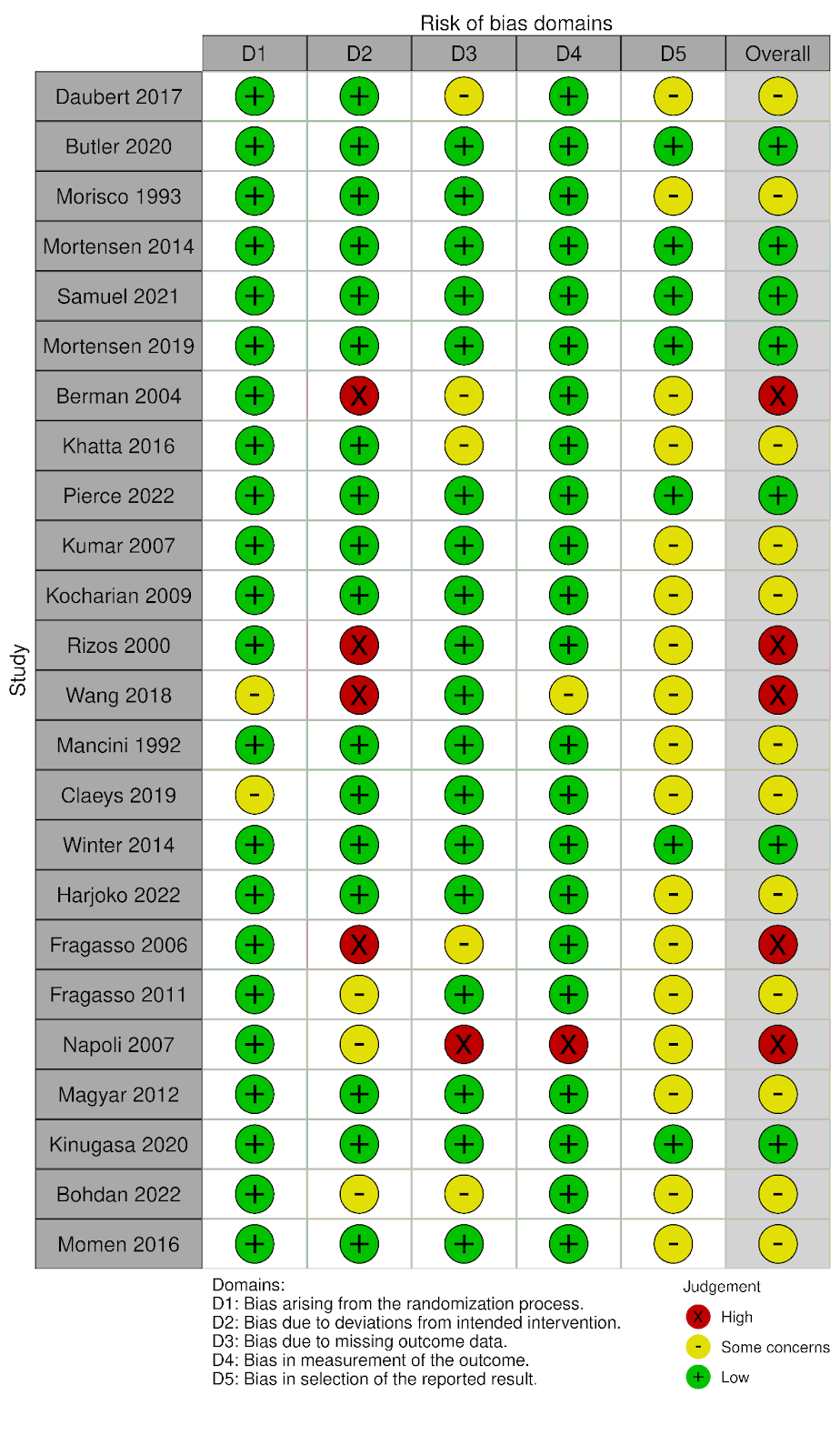


**Supplementary Figure S2: Forest Plots for Subgroup Analyses Based on Type of Therapeutic Agent**

A. Changes in Baseline LVEF


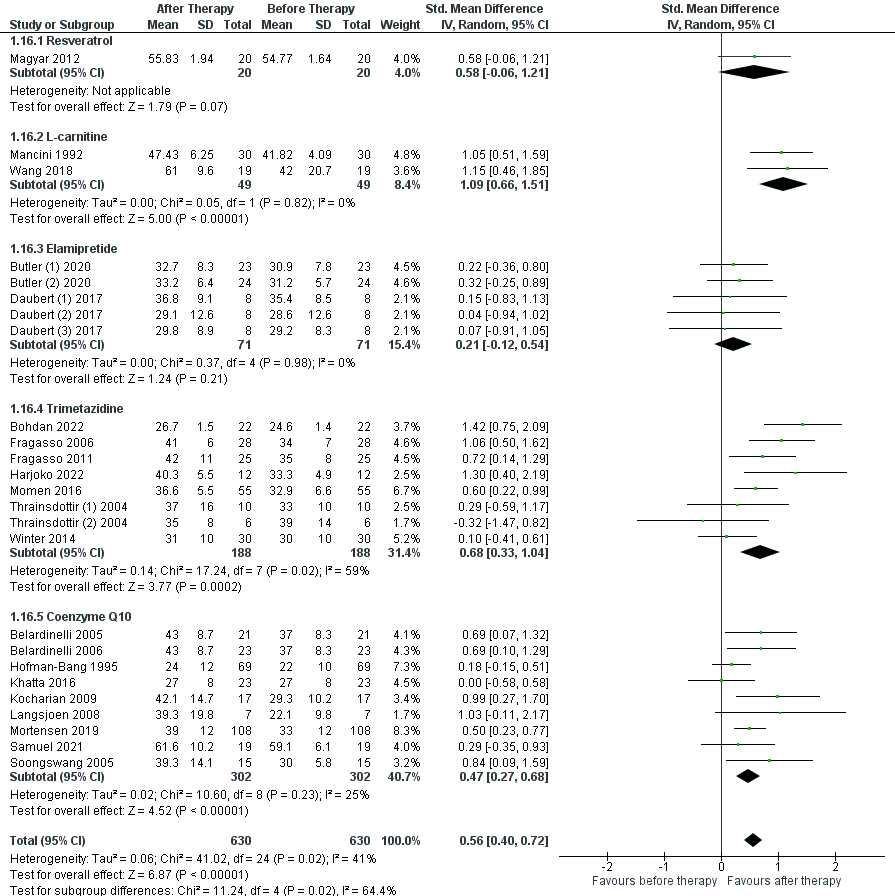


B. Improvement in NYHA Class

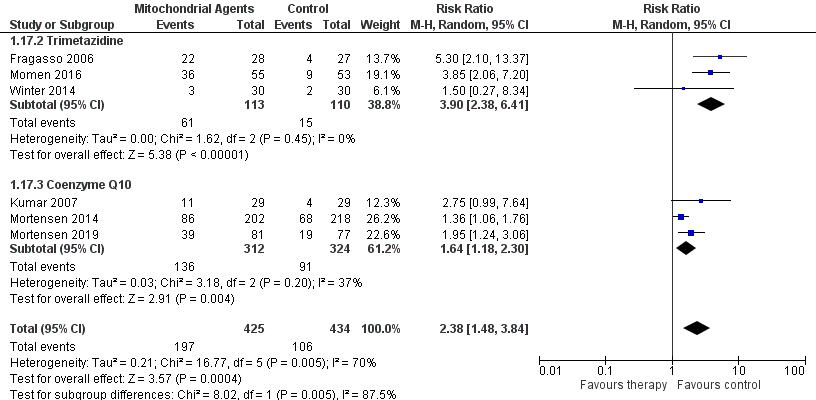


C. All-Cause Mortality


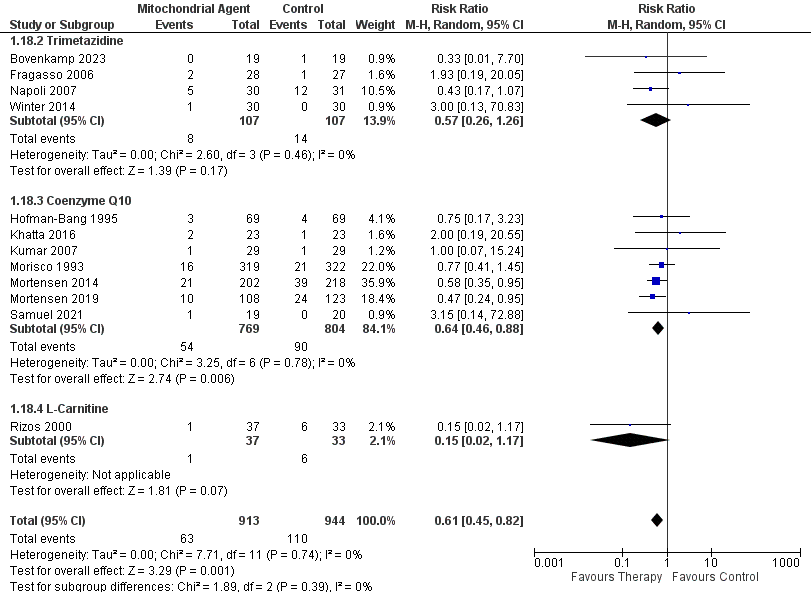


D. HF-related Hospitalization


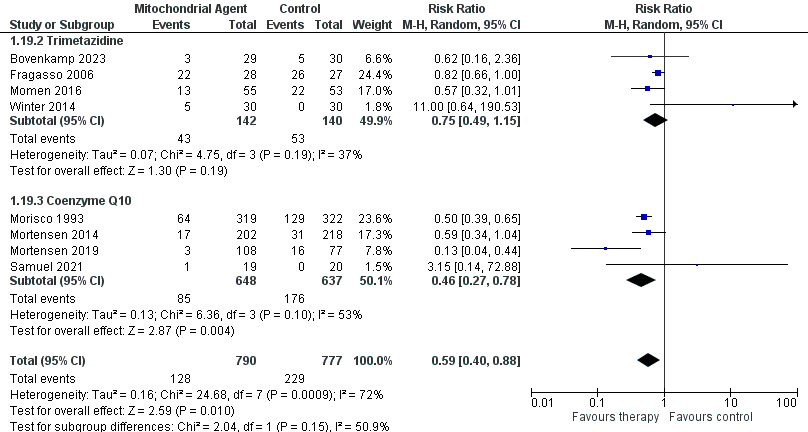


**Supplementary Figure S3: Forest Plots for Subgroup Analyses Based on Heart Failure Phenotype**

A. Changes in Baseline LVEF


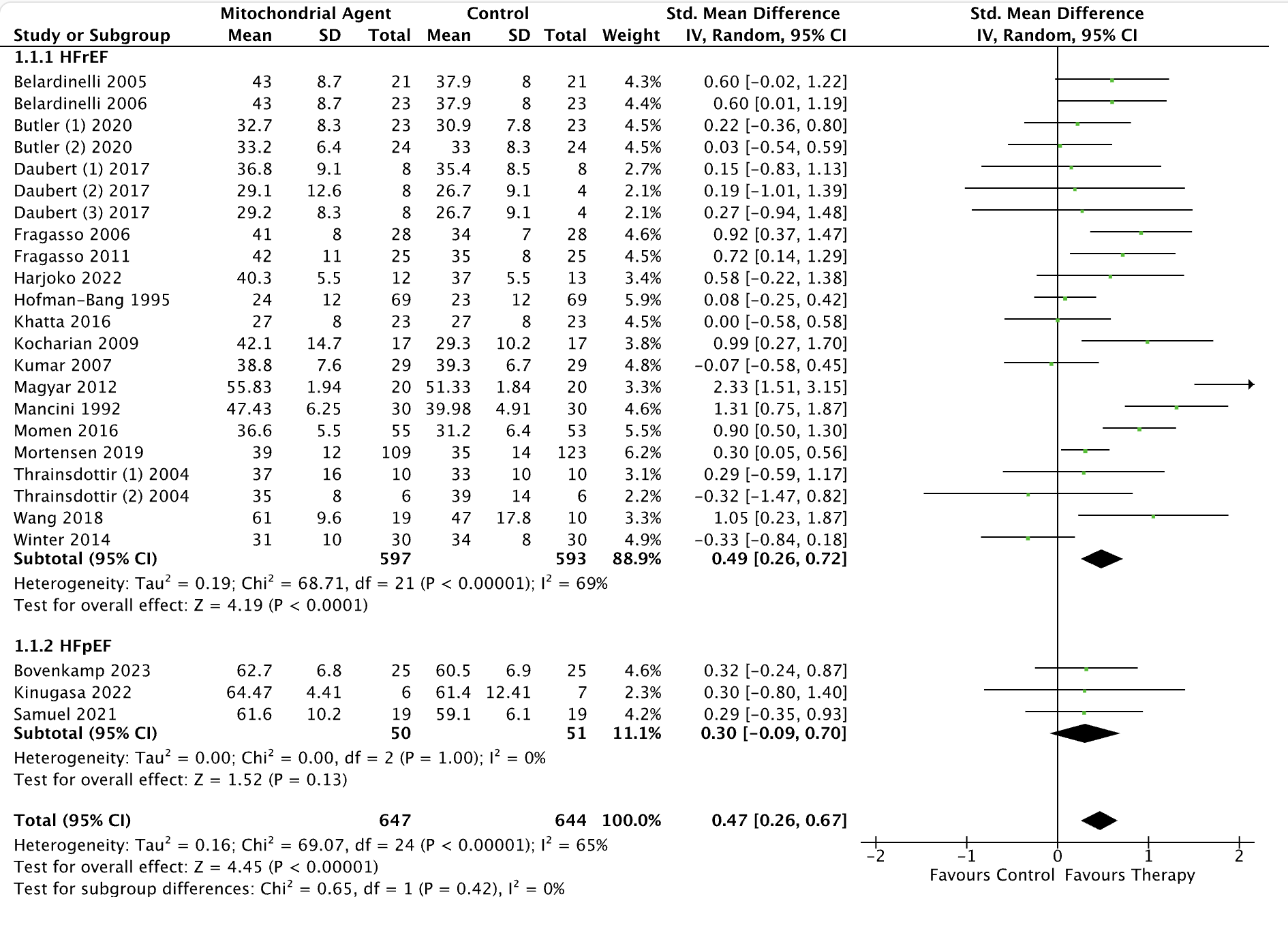


B. Changes in Baseline LVEF compared to Controls


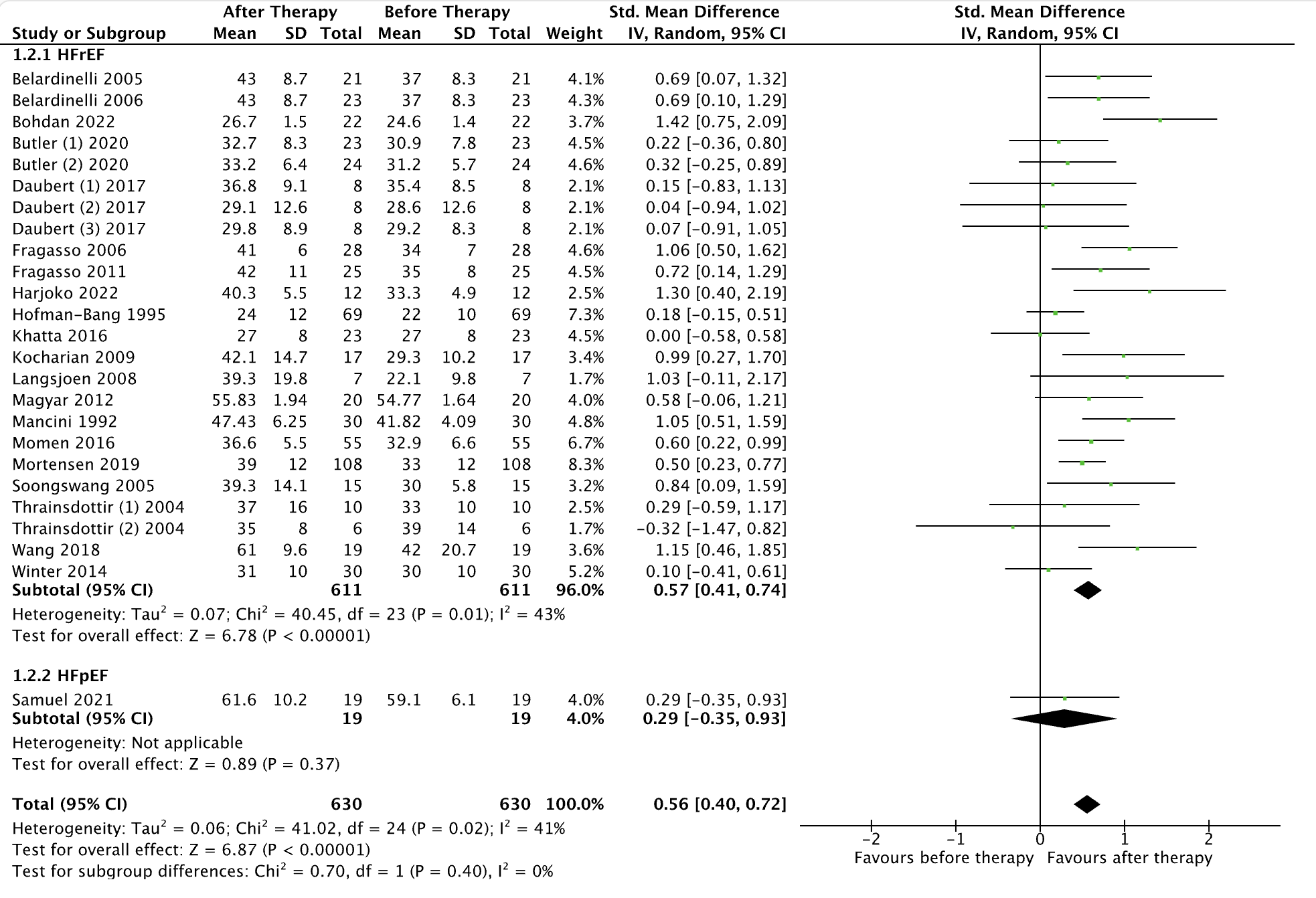


C. Change in Baseline Six Minute Walk Test


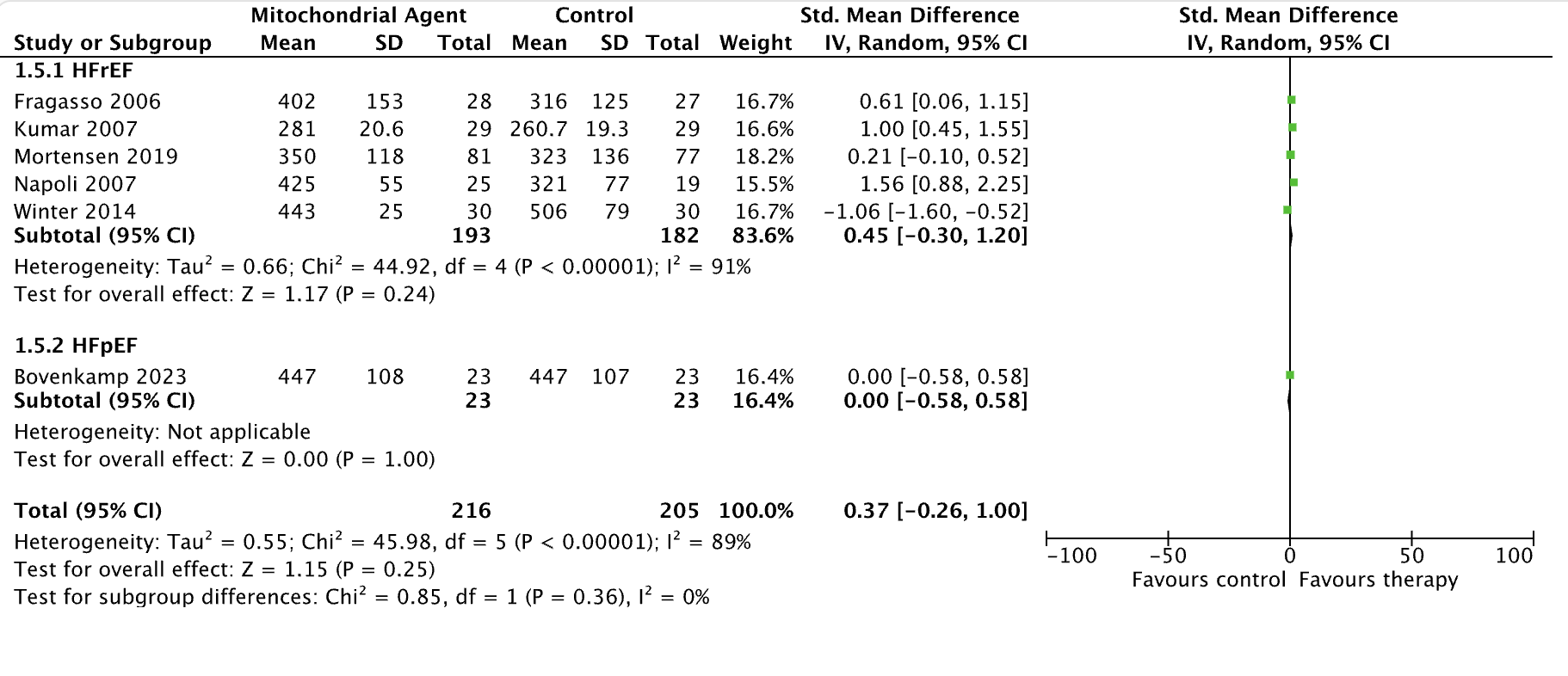


D. Change in Six Minute Walk Test compared to Controls


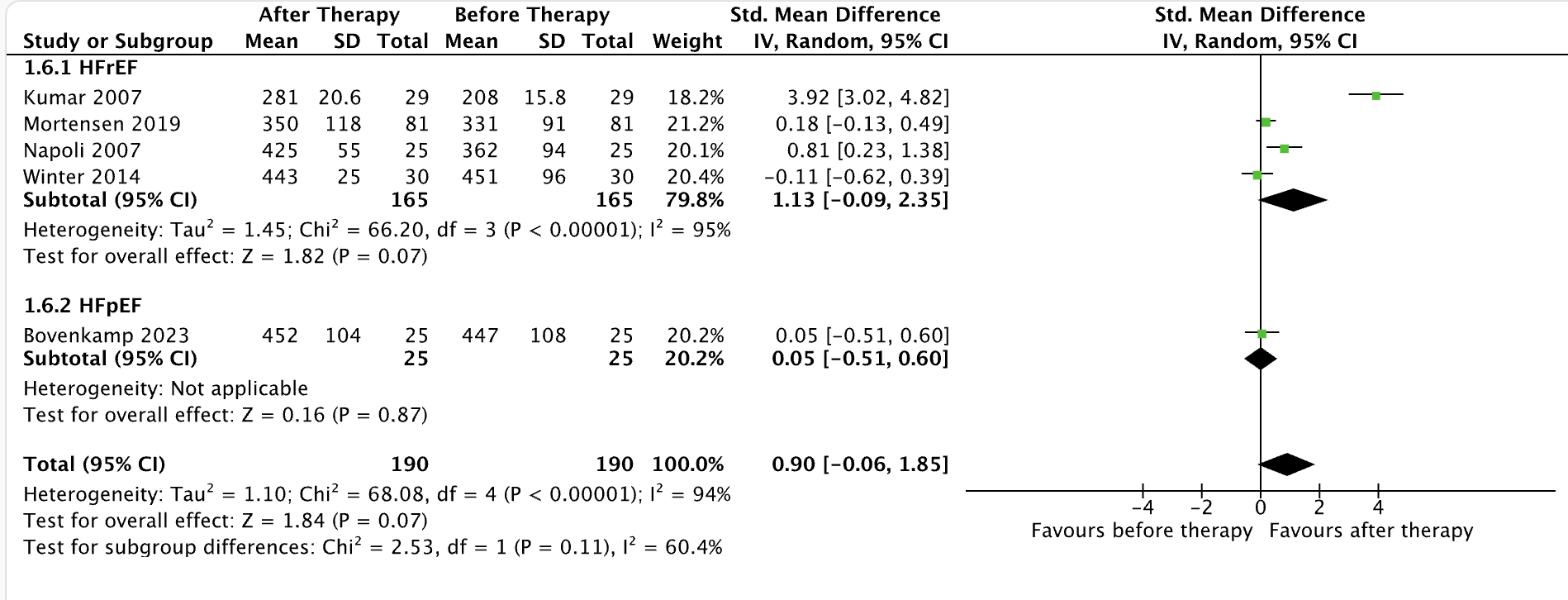


E. All-Cause Mortality


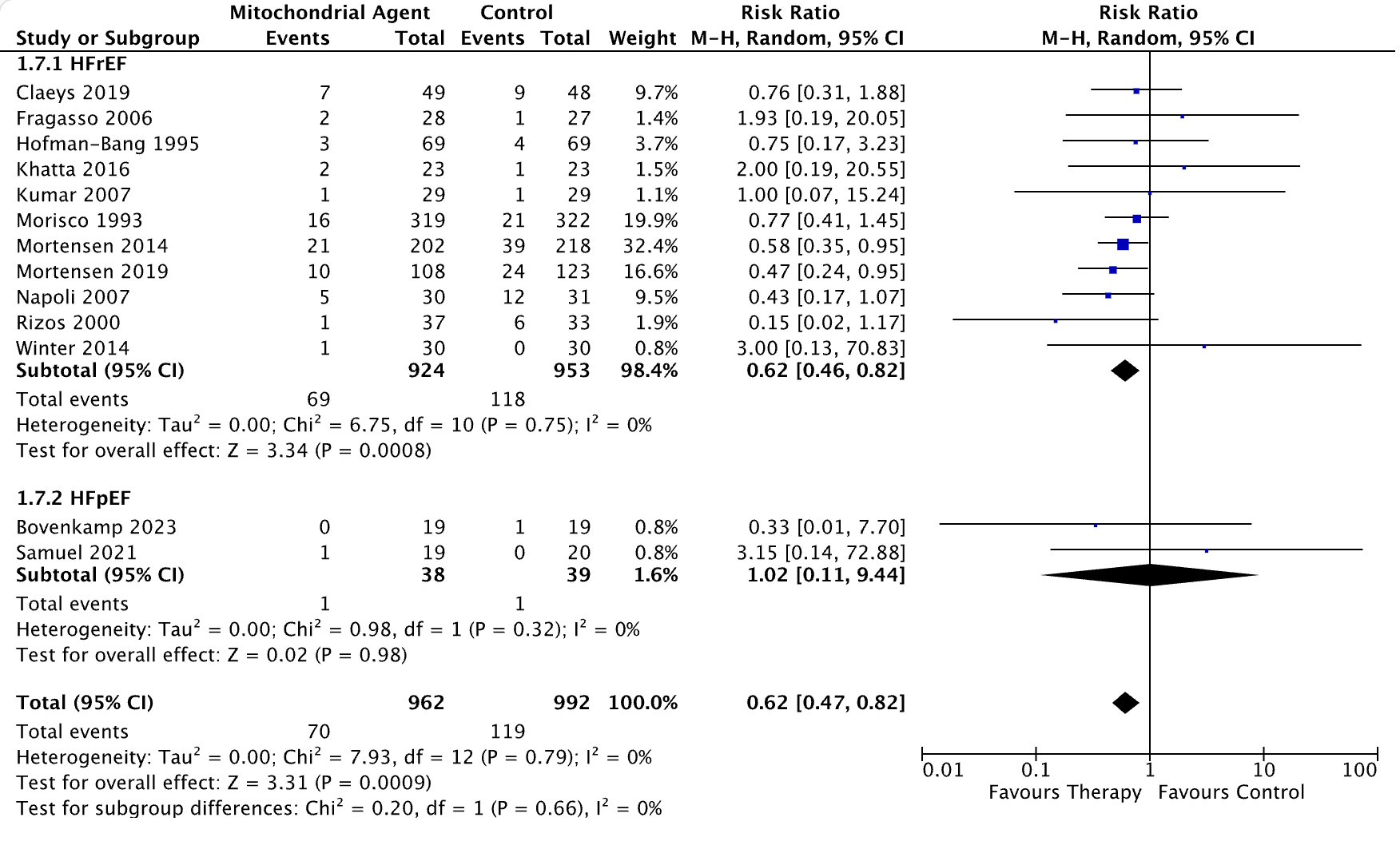


F. Heart Failure Hospitalizations


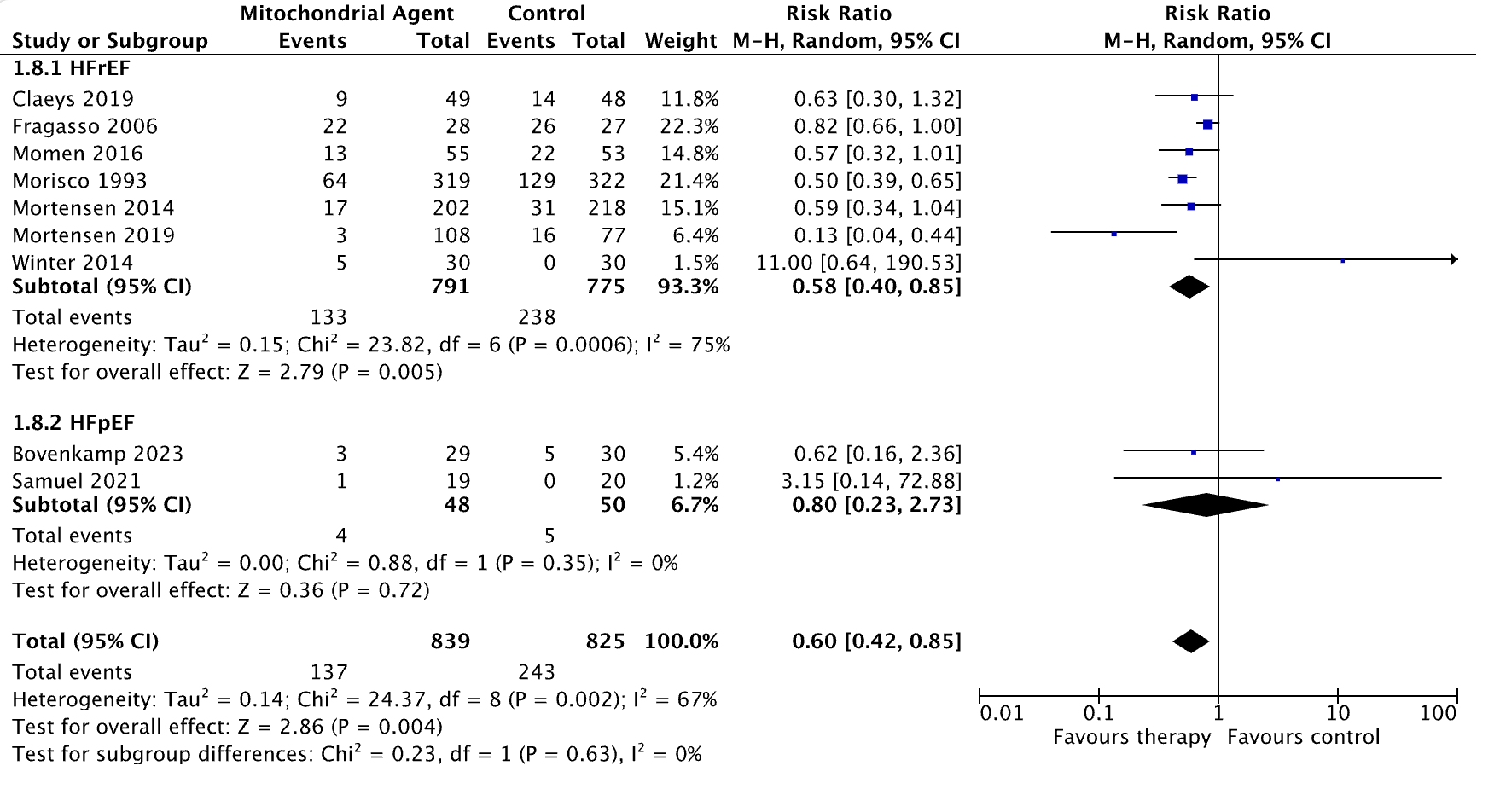


**Supplementary Figure S4: Forest Plots for Sensitivity Analyses Excluding Crossover Studies**

A. Changes in Baseline LVEF


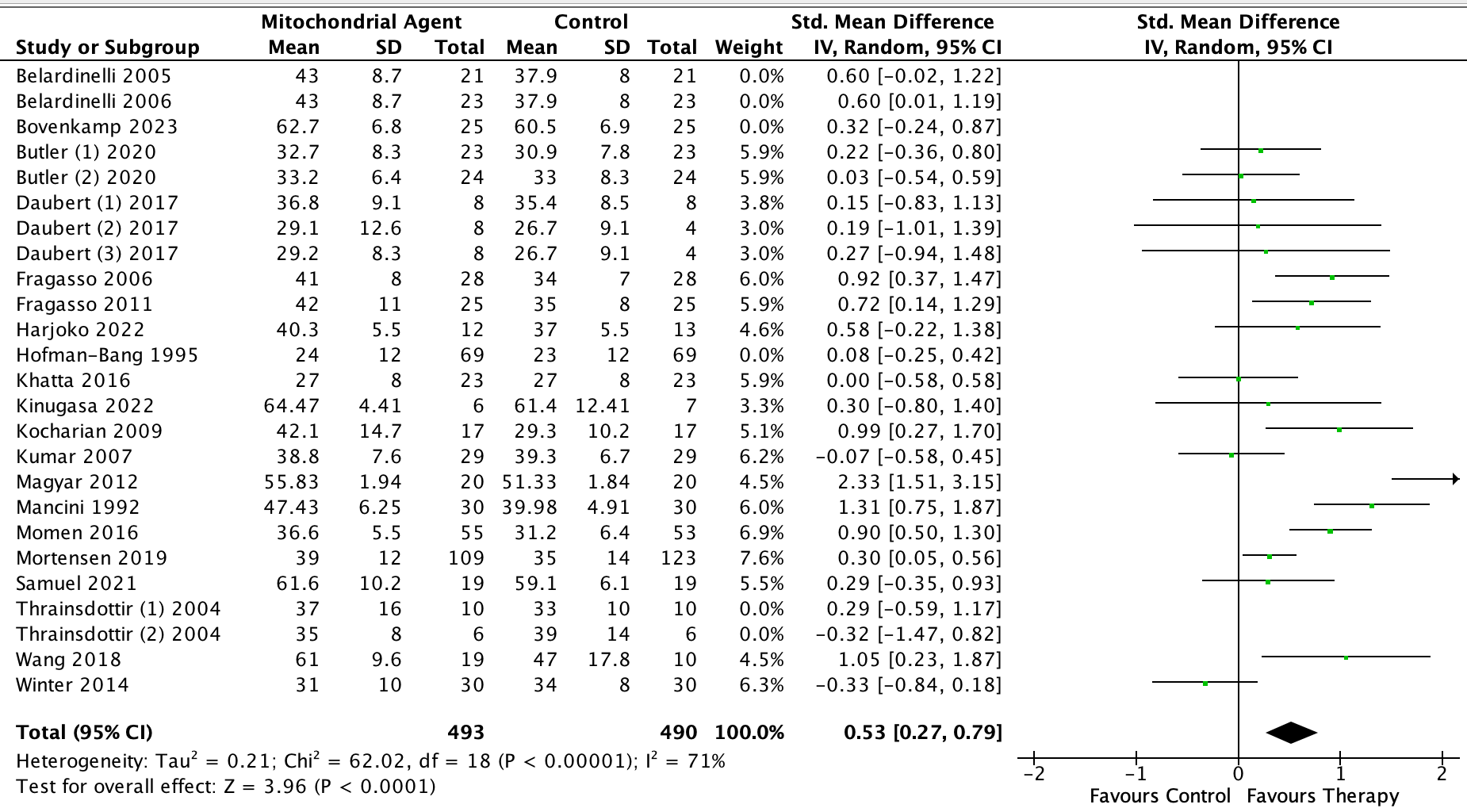


B. Changes in Baseline LVEF compared to Controls


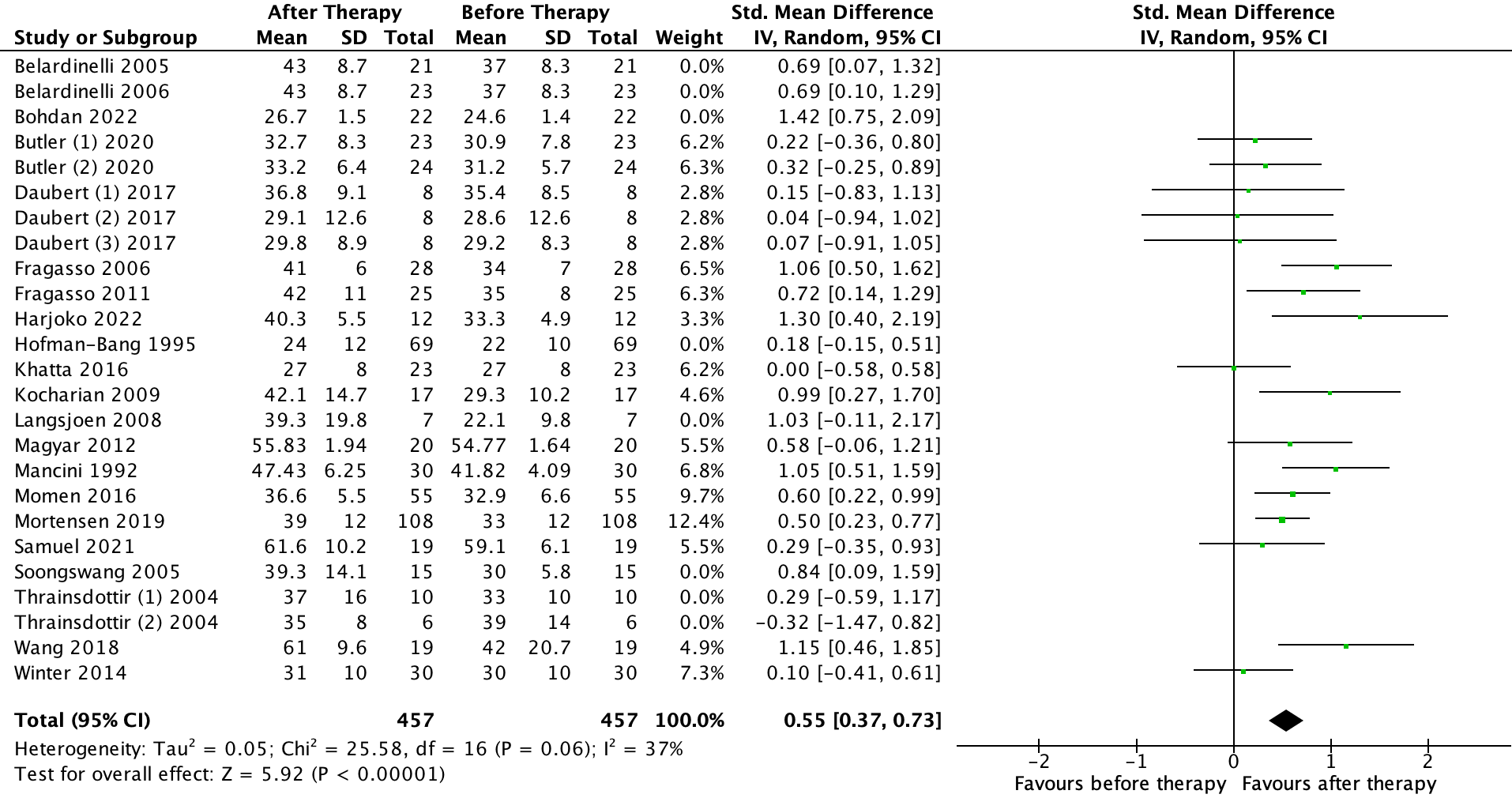


C. Change in Baseline Six Minute Walk Test


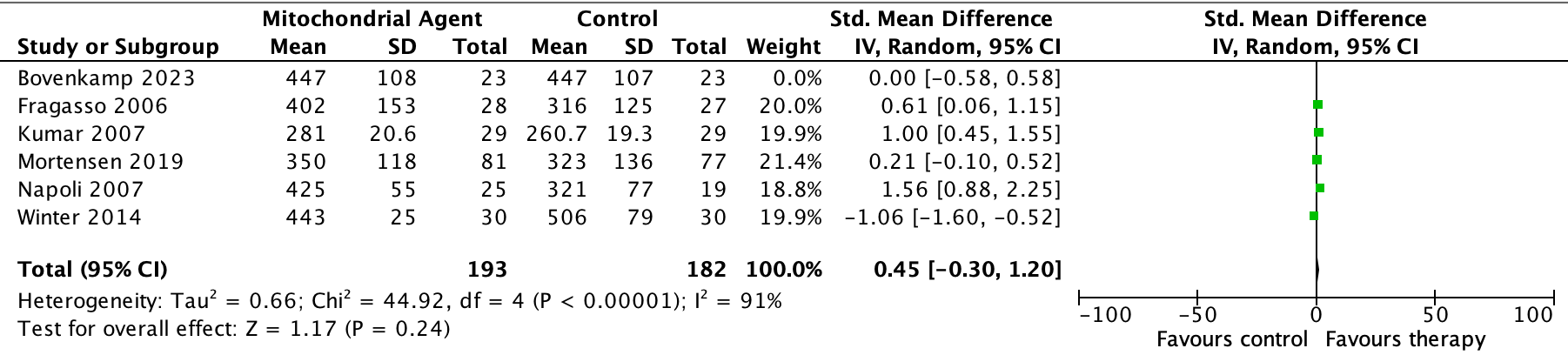


D. Change in Six Minute Walk Test compared to Controls


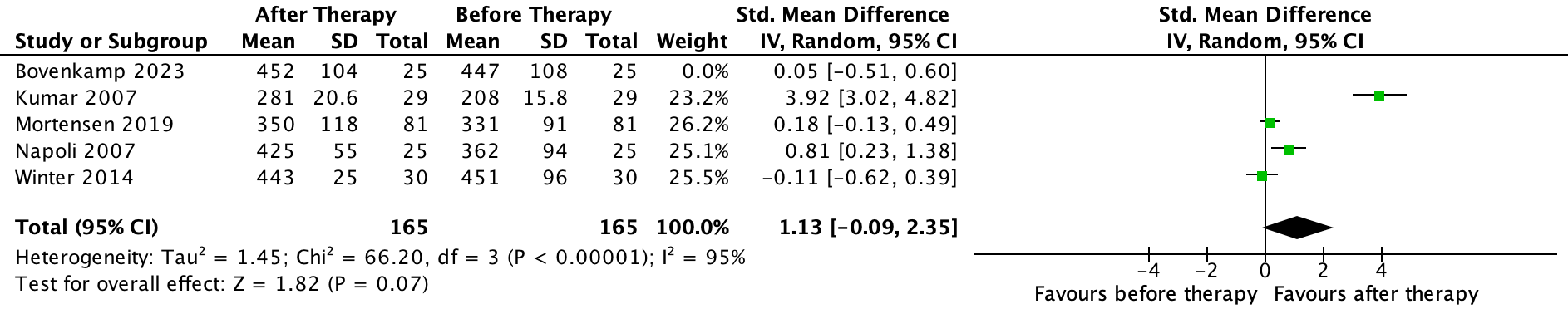


E. All-Cause Mortality


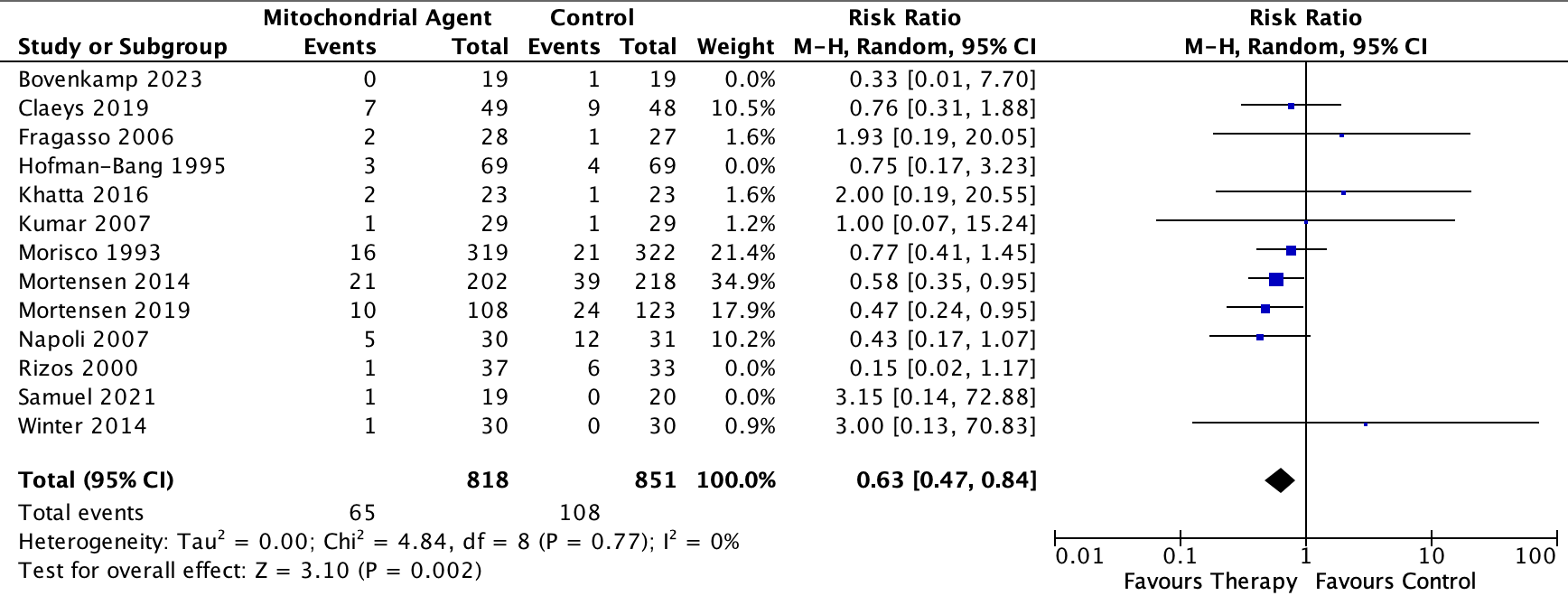


F. Heart Failure Hospitalizations


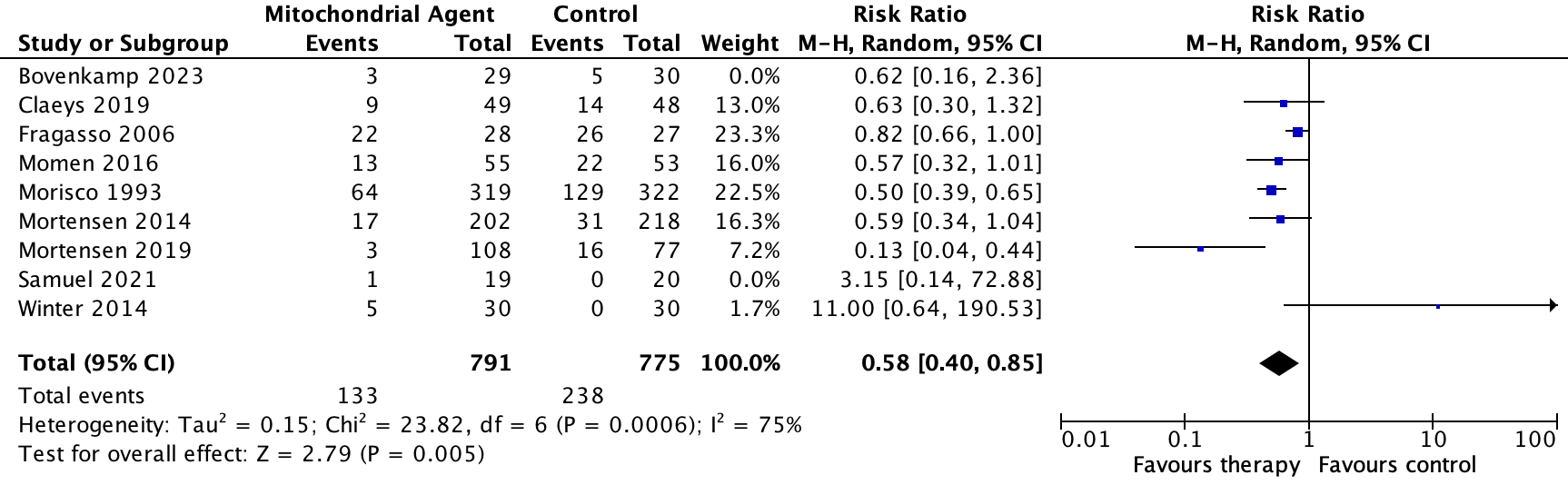


**Supplementary Figure S5: Meta-Regression Scatter Plot**


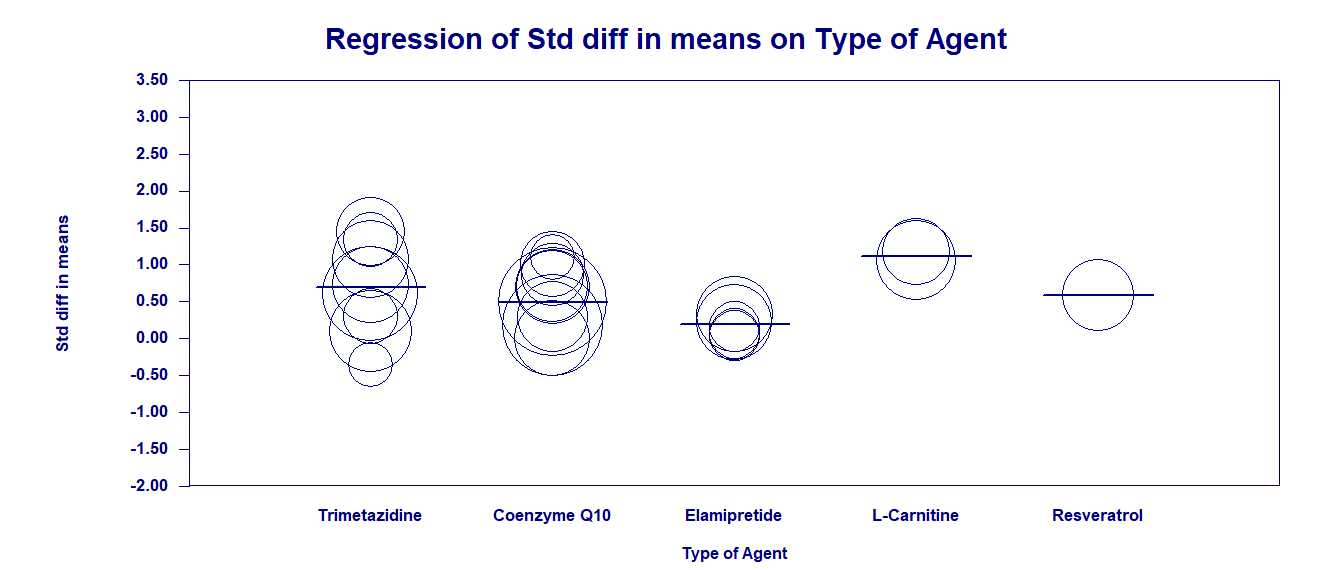


Figure S3 univariate meta-regression of change in LVEF across different mitochondrial agents. Each circle represents an individual study, with the size of the circle proportional to the study's weight in the analysis. The horizontal line within each group indicates the estimated mean effect size for that agent.

**Supplementary Figure S6: Funnel Plots for Publication Bias**

A. Change in LVEF compared to baseline (Eggers two-sided p-value=0.45)


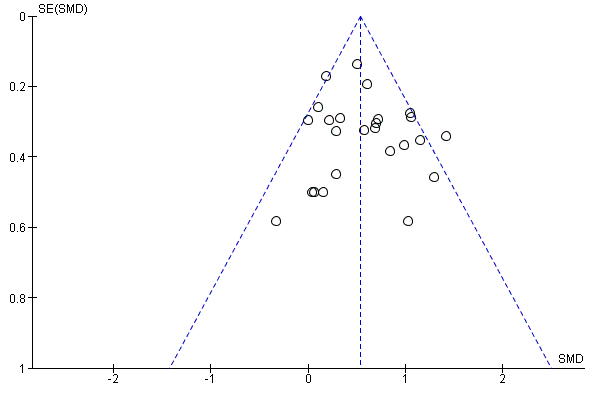


B. Change in LVEF compared to controls (Eggers two-sided p-value=0.33)


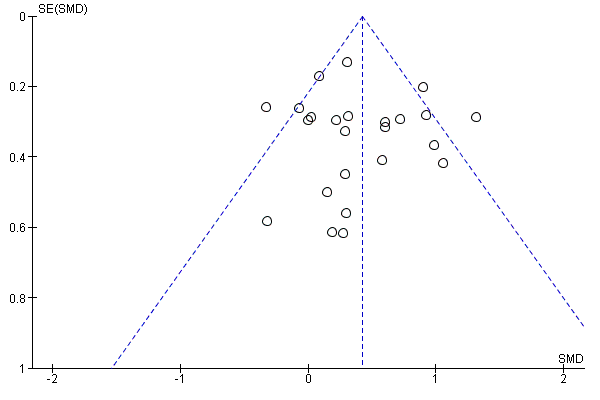


C. Mean NYHA Class


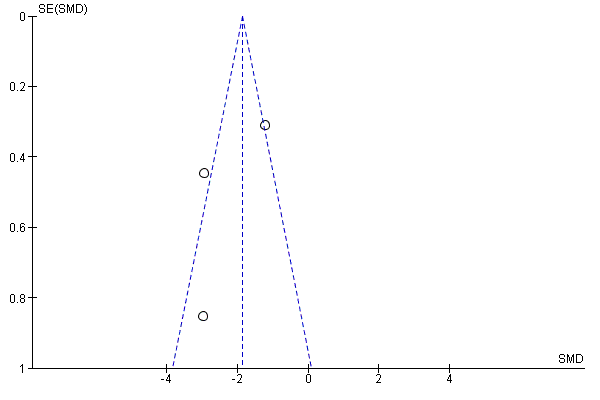


D. Improvement in NYHA Class


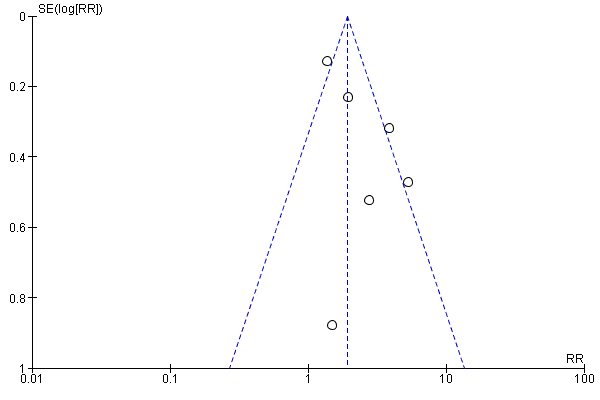


E. Six-Minute Walk Test Compared to Baseline Values


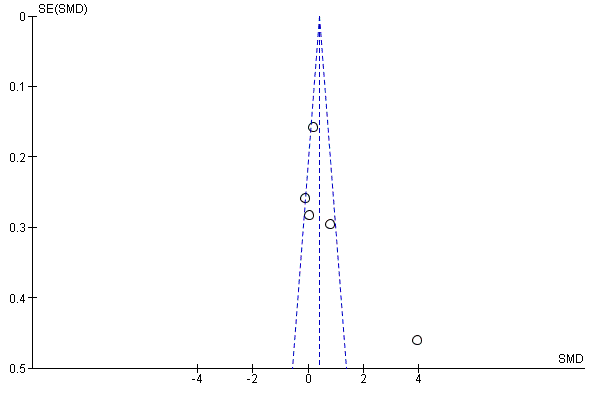


F. Six-Minute Walk Test Compared to Controls


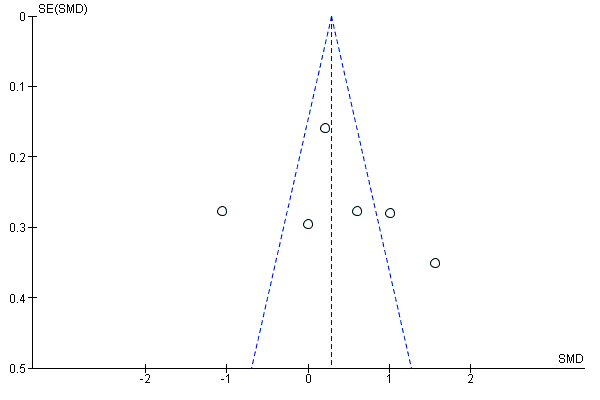


G. All-cause Mortality (Eggers two-sided p-value=0.28)


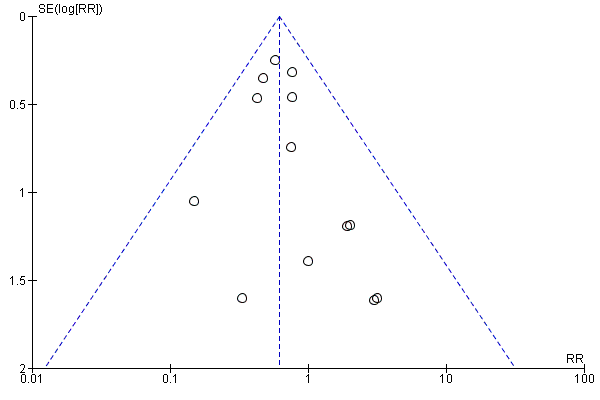


H. HF-related hospitalizations


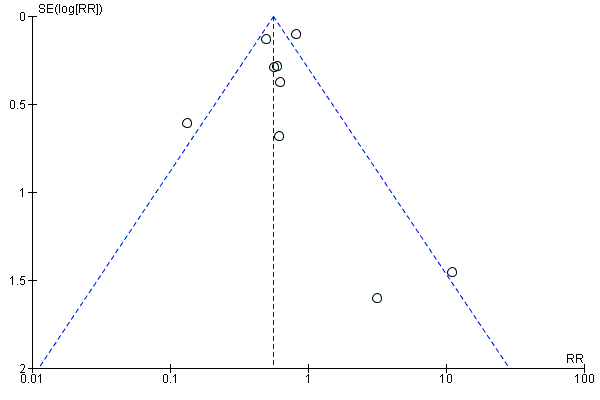

Supplement: Supplementary file 1 [file Supplemenatryfile1.docx]
